# Anthropomorphic Robotic Eyes: Structural Design and Non-Verbal Communication Effectiveness

**DOI:** 10.3390/s22083060

**Published:** 2022-04-15

**Authors:** Marko Penčić, Maja Čavić, Dragana Oros, Petar Vrgović, Kalman Babković, Marko Orošnjak, Dijana Čavić

**Affiliations:** Faculty of Technical Sciences, University of Novi Sad, Trg Dositeja Obradovića 6, 21000 Novi Sad, Serbia; scomaja@uns.ac.rs (M.Č.); spawn@uns.ac.rs (D.O.); vrgovic@uns.ac.rs (P.V.); bkalman@uns.ac.rs (K.B.); orosnjak@uns.ac.rs (M.O.); dijana.cavic@uns.ac.rs (D.Č.)

**Keywords:** social robots, healthcare, human-robot interaction, anthropomorphic robotic eyes, structural design, non-verbal communication, facial expression, emotion recognition, effectiveness

## Abstract

This paper shows the structure of a mechanical system with 9 DOFs for driving robot eyes, as well as the system’s ability to produce facial expressions. It consists of three subsystems which enable the motion of the eyeballs, eyelids, and eyebrows independently to the rest of the face. Due to its structure, the mechanical system of the eyeballs is able to reproduce all of the motions human eyes are capable of, which is an important condition for the realization of binocular function of the artificial robot eyes, as well as stereovision. From a kinematic standpoint, the mechanical systems of the eyeballs, eyelids, and eyebrows are highly capable of generating the movements of the human eye. The structure of a control system is proposed with the goal of realizing the desired motion of the output links of the mechanical systems. The success of the mechanical system is also rated on how well it enables the robot to generate non-verbal emotional content, which is why an experiment was conducted. Due to this, the face of the human-like robot MARKO was used, covered with a face mask to aid in focusing the participants on the eye region. The participants evaluated the efficiency of the robot’s non-verbal communication, with certain emotions achieving a high rate of recognition.

## 1. Introduction

In spite of the global fight against the coronavirus disease (COVID-19) [1], with over 3 million people contracting the virus daily [2], the experiences of healthcare workers of the most developed countries in the world [3,4,5,6,7,8,9,10] have shown that the healthcare system is fundamentally unprepared for a long-term or intense pandemic [11,12,13,14,15,16]. The system becomes overwhelmed quickly, risking the possibility of many people receiving substandard care [17,18,19,20,21,22], with the rate of diagnosis of diseases with the highest mortality rates, such as malignant [23,24,25,26] and cardiovascular [27,28,29,30] diseases, dropping substantially. Both those fallen ill in the pandemic and those with chronic conditions require medical care based on interpersonal interaction, which is neither easy nor safe to provide during pandemic conditions [31,32,33,34,35]. Keeping in mind the numerous mutations of the virus, and new, more contagious variants [36,37,38,39], in spite of social distancing [40,41,42,43], preventative measures [44,45,46,47], and vaccination efforts [48,49,50,51], it is assumed that the use of disruptive technologies such as industry 4.0 [52,53,54,55,56], internet of things (IoT) [57,58,59,60,61], internet of medical things (IoMT) [62,63,64,65,66], and others [67,68,69,70,71], together with robotic technologies [72,73,74,75,76], could have a key role in the fight against the pandemic and in relieving the healthcare system as well as preventing its collapse on a global scale.

According to Ref. [77], examples of notable uses of disruptive and robotic technologies in the fight against the pandemic and the preservation of public health are seen in: (i) diagnosis robots for fast scanning, and mass testing of people by measuring body temperature and taking oropharyngeal swabs, (ii) logistics robots for safe transport of infective waste, sterilized medical material, swab samples, blood and urine (iii) healthcare robots meant for simple tasks such as transporting food and medication to patients as a key measure against infection transmission, (iv) disinfection robots for cleaning and disinfecting hospital and public spaces with the goal of reducing the frequency of human-human interaction (HHI), and (v) socially assistive robots (SARs) for human-robot interaction (HRI) as a strong support measure for the healthcare system in the fight against the pandemic and maintaining social distance. It should be noted that robots do not require face masks, can be disinfected quickly and easily and, most importantly, cannot get sick. On the other hand, using a face mask invokes feelings of empathy in people, and reduces anxiety [78,79,80], so SARs can wear face masks which is in accordance with socially responsible behavior.

Based on the conducted research and available praxis examples [81,82,83,84,85,86], it is assumed that the social and psychological aspect of medical care can be supplemented with the use of SARs. For SARs to function successfully in an everyday human environment, HRI is key [87,88,89]. Aside from verbal communication, it is of great importance to also realize non-verbal communication, which can communicate a lot of information in a very short period of time—in a social context, 60–65% of the intended meaning is conveyed through non-verbal behaviors [90,91,92]. Although posture and gesticulation have a role, the most expressive is by far the face, with the eyes and eyebrows being the most expressive part of the face, representing a powerful tool for showing emotion, especially today, during the pandemic, where a large part of the face is often covered by a face mask.

The capabilities of a robotic system, such as visual attention models and facial and object recognition systems, allow robots to establish and maintain eye contact with a conversation partner, giving the impression of a focused and more natural interaction. The possibility of eyebrow and eyelid position adjustment enables the generation of facial expressions with the goal of simulating different emotional states. Blinking and the speed of eyeball and eyelid movements are both important functionality aspects, which contribute to the perception of the robots’ movements as natural. If cameras are built into the eyeballs of the robot, it becomes possible to use vision systems to realize certain functions of artificial sight, such as facial or object detection and distance assessment relative both to the robot and others, all of which enables a wider spectrum of assistive tasks the robot can complete. Body language, gesticulation, and facial expressions are important aspects of the functionality of socially interactive robots. Since the eyes are the most expressive part of the face—especially if the face is rigid and motionless, which is the case with most robots, special attention should be paid to the development of an appropriate eye structure for the robot.

The primary goal of this paper is the biomimetic design of a mechanical eye system, with adequate kinematic characteristics, which should functionally provide a spectrum of movements that coincide with the natural movements of human eyes and eyebrows, allowing the robot to simulate emotional states. The proposed mechanical system represents an adequate hardware platform for the development and implementation of robotic vision and algorithms with different purposes, such as face detection, facial recognition, emotion recognition, etc. By using a high-quality vision system, based on a sophisticated mechanical and control system, robots can potentially relieve the healthcare system, contributing to the quality of care of sick and threatened individuals, as well as the safety of healthcare workers.

The paper is structured as follows: the first section describes the motivation and goal of the research; the second section shows the structure and kinematics of the human eye; the third section analyzes the state-of-the-art, focusing on two groups of problems; the fourth section explores the problems discussed in this paper in detail; the fifth section shows the structure of the mechanical systems of the eyeballs, eyelids, and eyebrows; the sixth section proposes the architecture of a control system for the eyes and eyebrows; the seventh section explores the ability of the proposed mechanical system to realize non-verbal communication; a summarization and discussion of the results is presented in the eight section; finally, the ninth section contains the conclusion and possible directions for future research. It should be noted that each section contains a short summary.

## 2. The Human Eye

Aside from their primary role—sight, the eyes, as well as the eyebrows, are key components for facial recognition and non-verbal communication. Depending on how open the eye is, the position of the eyeballs, eyelids, and eyebrows, as well the speed of their movements, different emotions are expressed. Due to this, special attention was paid to the structure and kinematics of the eye.

### 2.1. Structure

**Figure 1:**
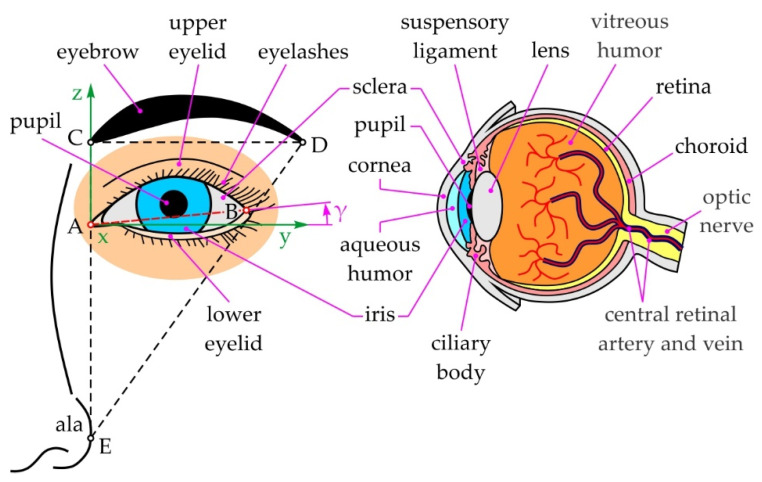
Human eye—structure and auxiliary elements.

The eyes are the most important sensory organ in humans, enabling visual perception of the surrounding world—close to 80% of all impressions of the outside world are perceived by sight [93,94]. Every second, the eyes are adapting to their surroundings, the light, colors, and various other effects, absorbing information and forwarding it to the brain [95,96]. According to Refs. [97,98], the eye apparatus contains: (i) the eyeball, (ii) the visual pathways, and (iii) the auxiliary elements of the eye (see Figure 1).

The eyeball, thanks to the structure of its media, the dioptric apparatus and presence of neuroepithelial elements in the retina, enables the reception of visual impressions. The visual pathways connect the neural membrane of the eyeball—the *retina*, with the visual centers of the brain. Therefore, a visual stimulus formed on the retina is transported to the relevant centers in the brain for further interpretation of the signal. The auxiliary elements of the eye are the eyebrows, eyelids and eyelashes, the lacrimal apparatus, the ocular muscles, the orbital cavity, and others. Their primary function is both to protect the eyeball and enable all the complex processes the eye performs daily. The eyeball is akin to a sphere and consists of 3 mantles and a gelatinous filling that makes up 4/5 of the eyeball. The front-facing part of the outside mantle is the *cornea*—an integral part of the dioptric apparatus due to its transparency and slight curvature, while the back-facing part is opaque, significantly thicker, and white in color, called the *sclera*. The middle mantle, its main function being to feed the eyeball, encompasses the *iris*—the diaphragm regulating the amount of light intake, the *ciliary body*—it produces and secretes the *aqueous humor*, and the *choroid*—a key element in the feeding of the optical part of the retina. The inner mantle—the *retina*, in an embryonic sense represents an extension of the brain matter, and thanks to the presence of neuroepithelial cells, enables the reception of visual impressions. The inside of the eyeball encompasses the *aqueous humor*—and clear and completely transparent liquid which is the main factor determining intraocular pressure, the *lens*—a transparent biconvex structure for the refraction of light, and the accommodation and absorption of ultraviolet (UV) rays, and the *vitreous humor*—a thick, viscous, transparent structure which provides the eyeball with stiffness. It should be noted that the *optic nerve*, as a part of the visual pathways, connects the cells of the retina with the apparatus situated in the brain by sending impulses.

The eyebrow, with its structure and arched shape, protects the eye from excess light, and prevents water and sweat from flowing into the eye. Except for their protective role, the eyebrows have a major role in everyday communication and are as important as the eyes in the context of facial recognition [99,100]. The eyelids are thin skin formations made from muscles and connective tissue that close the orbital cavity from the front, protecting the eyeball. When the eyelids are open, they form an ellipsoidal opening—due to the lacrimal apparatus, the inner corner of the opening is rounded (point A, i.e., medial canthus), while the outside corner is sharp (point B, i.e., lateral canthus). Visually, an important characteristic of the eye is the canthal tilt (CT), the angle between the y-axis and the line connecting the medial and lateral canthus (see Figure 1, angle γ). The value of this angle depends on the sex and ethnicity of the person [101,102], with a positive CT being a characteristic of a female eye, while a male eye has a neutral or slightly positive CT. It should be noted that a positive CT greatly influences the perception of a face as attractive and youthful [103,104], making CT correction one of the most often undergone aesthetic procedures in modern society [105,106]. By blinking, the eyelids help the drainage of tears which take with them impurities from the front surface of the eye. Along the edges of the eyelids are the eyelashes, and the denser they are, the more protection they offer both from dust and mechanical injuries. The lacrimal apparatus keeps the eye moist, thus ensuring the transparency of the retina. Furthermore, the biochemical characteristics of the tears protect the eye from injuries and infections. The conjunctive connects the eyelids with the eyeball, while the ocular muscles position the eyeball within the orbital cavity. These muscles—a total of 6, with 2 angled and 4 straight, allow the eyeball to rotate in all directions. The orbital cavity is a pyramid-shaped cavity whose walls are formed by different bones, providing further protection for the eyeball [97,98]. The diameter of the eyeball is approximately 25 mm, with no notable difference between sexes and age groups [107]. However, the pupillary distance (PD) differs between men and women, equaling, on average, 65 mm and 61 mm, respectively [108].

### 2.2. Kinematics

**Figure 2:**
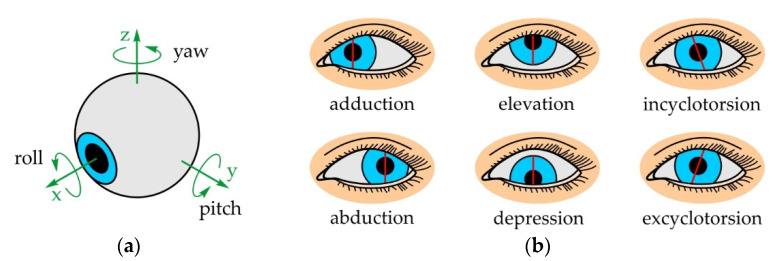
(**a**) Eyeball rotations; (**b**) Types of eyeball movements depending on the rotation direction.

The eyeball has 3 degrees of freedom (DOFs) allowing the rotation around all three axes (see Figure 2a): around the z-axis or yaw rotation, around the y-axis or pitch rotation and around the x-axis or roll rotation. Depending on the direction of the rotation (±), there are different types of movements (see Figure 2b): adduction and abduction enable horizontal rotation of the eyeball around a vertical axis, shifting the gaze medially (toward the nose) and laterally (away from the nose), respectively; elevation and depression refer to the vertical rotation of the eyeball around a horizontal axis, shifting the gaze upward and downward, respectively; incyclotorsion and excyclotorsion allow the rotation of the eyeball around the line of sight, moving the eyeball medially and laterally, respectively.

According to Ref. [109], the ranges of motion of adduction and abduction are nearly the same and equal, 44.9 ± 7.2° and 44.2 ± 6.8°, respectively, so the total yaw range of motion of the eyeball equals approximately 90°. On the other hand, the ranges of motion of elevation and depression differ and equal, 27.9 ± 7.6° and 47.1 ± 8.0°, respectively, making the total pitch range of motion approximately 75°. The smallest range of motion, if afforded to incyclotorsion and excyclotorsion, are only a few degrees each [110], which is why the roll motion of the eyeball is disregarded in this paper. The speed of the eyeball depends on the type and nature of the motion, and is determined by observing both eyes simultaneously. According to Refs. [111,112], the principal types of eye motion are: (i) saccades, (ii) smooth pursuit movements, (iii) vergence movements, and (iv) vestibulo-ocular movements. Horizontal and vertical saccades are rapid movements of the eyes between fixed points that abruptly shift the direction of the gaze—for example, reading a newspaper or scouring the objects in a room, and in this case, the angular speed of the eyeball reaches values of 400–800°/s. On the other hand, smooth pursuit movements are gentle and very slow movements of the eyes that enable the tracking of objects in motion at great distances, and in this case, the angular speed does not exceed 30°/s. Differing from these types of movement where both eyes rotate in the same direct, vergence movements rotate the eyeballs in different directions allowing them to focus on specific objects—for example, when moving a finger to and from the nose, and in this case, the angular speed reaches values of 30–150°/s. Vestibulo-ocular movements are reflexive eye movements that compensate sudden and abrupt head movements to stabilize the image seen by the eyes, and in this case, the angular speed reaches values of 800°/s.

Blinking is a complex, short, and nearly periodic physiological action during which the eyelids fully close and fully open, while the duration depends on the type of motion. According to Refs. [113,114], the principal types of eyelid movements are: (i) reflex blinking—involuntary, abrupt, and rapid movements caused by stimulation of the retina, for example, a touch or any other peripheral stimulus; the duration of this type of blink is the shortest and equals 205 ± 18 ms, (ii) voluntary blinking—movements which the subject does willingly due to internal or external commands; the duration of these movements is longer and equals 275 ± 37 ms, and (iii) spontaneous blinking—unconscious and continuous movements with the longest duration of any type that equals 334 ± 67 ms. It should be noted that the closing phase lasts 2.5 times less than the opening phase, meaning that the speeds differ as well [114]. The spontaneous blink rate is on average 10–20 blinks/min [115], depending on age, gender, time of day, as well as the fatigue and concentration of the subject—women blink twice as much as men in the same time period [116]. According to Ref. [117], the range of motion of the upper eyelid depends on the type and phase of the motion, with the highest value being reached during the closing phase of reflex blinking, 41.3 ± 5.3°, and with the angular speed of the upper eyelid reaching values of 1108.0 ± 157.0°/s. The range of motion of the lower eyelid has only been discussed in the available literature as a consequence of vertical saccades, when the eyelids move together with the eyeball in an up-and-down motion [118]. Visually, when in the normal eyelid position, the upper eyelid is 2 mm bellow the periphery or the iris, while the lower eyelid is exactly on the periphery of the iris [119]. On the other hand, some authors measure the distance between the eyelids and the center of the pupil [120,121]. Observation has shown that the line of contact between the eyelids when the eyes are closed is between the center of the pupil and its periphery, which is when the lower eyelid achieves the maximum possible rotation angle.

According to Ref. [122], the ideal position of the eyebrow is defined by a right-angle triangle (see Figure 1) formed by the medial and lateral canthus (points C and D, respectively), and the outside part of the nose, the *ala* (point E). There are 7 principal types of eyebrow movement [123] with their amplitudes directly depending on which part of the eyebrow is being actuated (medial, above the pupil or lateral) and in which direction (raising or lowering). According to Ref. [124], eyebrow raising ability decreases with age, so for the age group 20–39 and ≥40, the amplitude equals 13.0 ± 2.9 mm and 9.8 ± 2.0 mm for the medial canthus, and 15.7 ± 2.6 mm and 12.7 ± 1.7 mm for the midpupillary line, respectively. On the other hand, for markers placed on the midpupillary line and for voluntary movements, the maximum raising amplitude for the left and right eyebrow equals 9.75 mm and 10.14 mm, with the angular speeds reaching 24.11 mm/s and 25.87 mm/s, respectively [125]. However, during abrupt movements caused by fear, the eyebrows, along with the upper eyelids, raise reflexively, and in this case, the speed of the eyebrows is much higher.

### 2.3. Summary

Based on the structure and kinematics of the eye and eyebrows, the following is concluded: (i) although the eyeball has 3 DOFs, in this instance, only the pitch and yaw movements are relevant, with their range of motion equaling approximately 90° (adduction 45ׄ° + abduction 45ׄ°) and 75° (elevation 25° + depression 50°), respectively; the roll motion of the eyeball has a very small range of motion, and is thus disregarded; the angular speed of the eyeball reaches its highest values during saccadic and vestibulo-ocular movements—nearly 800°/s, and its lowest during smooth pursuit movements, not exceeding 30°/s; (ii) the kinematic parameters of the upper and lower eyelids are different—the upper eyelid is nearly two times wider that the lower eyelid, so its range of motion is also twice as big, and accordingly, the angular speed as well; the maximum ranges of motion of the upper and lower eyelids equal 45° and 20°, respectively, with the angular speed of the upper eyelid during the closing phase of reflexive movements reaching approximately 1100°/s; it should be noted that the closing phase lasts around 2.5 times less than the opening phase, with the total duration of a blink being 0.2–0.4 s; (iii) the kinematics of the eyebrows are complex and depend on the part of the eyebrow being actuated as well as the direction; the amplitude when raising the eyebrows is approximately 10–15 mm, with the speed during voluntary movements reaching 25 mm/s; however, during reflexive movements of the eyelids and eyebrows caused by fear, higher speeds should be expected.

## 3. State of the Art

The literature review should provide information on realized robots that are able to intuitively and transparently express human-like emotions by moving characteristic parts of the face, such as the eyes, eyebrows, and mouth, independently of the rest of the face. Accordingly, there are two approaches in the design and realization of socially interactive robot faces. The first refers to rigid faces with moving mechanical parts—eyeballs, eyelids, eyebrows, and mouth, while the second approach involves a rigid face on which the eyes, eyebrows, and mouth are displayed using light-emitting diodes (LEDs). However, it is possible to combine these two approaches. Accordingly, the literature review will cover two basic groups of problems: (i) robots that have rigid faces and moving mechanical parts such as eyeballs, eyelids, eyebrows, and (ii) robots that also have rigid faces, where the eyeballs and eyelids actuate mechanically, while the eyebrows and/or mouth are displayed using LEDs. We will additionally analyze: (i) the number of DOFs of the eyes and eyebrows, because a larger number of DOFs allows a wider range of movements and, consequently, a wider range of non-verbal facial expressions—emotions, (ii) how motion is transmitted from the actuators to the eyeballs, eyelids, and eyebrows—output links of the driving mechanisms, (iii) types of actuators and sensors used, and (iv) ability of the robots to produce facial expressions.

### 3.1. Rigid Robot Face with Moving Mechanical Parts

A humanoid robot head called HYDROïD with minimal emotion capabilities is shown in [126]; the robot has 4 DOFs eyeballs, 3 DOFs eyebrows, and a 5 DOFs mouth mechanism; pitch and yaw movements of the eyeball are enabled by gear and pulley systems, respectively, while the eyeballs and eyebrows are actuated by Athlonix 12G88 motors and GWS Naro servomotors, respectively; the robot is capable of producing 2 facial expressions (happiness and sadness).

A robotic head called EMYS (EMotive headY System) with emotion expression capabilities is shown in [127]; the robot has 2 DOFs eyeballs and 4 DOFs eyelids actuated by micro Hitec HS-65HB servomotors, while the Logitech Sphere AF color complementary metal-oxide semiconductor (CMOS) camera located in the nose, Kinect motion sensor, as well as a microphone for sound reception and speech recognition are used to perceive the environment; the robot is capable of producing 6 facial expressions (anger, disgust, fear, joy, sadness, and surprise).

A multi-sensor robotic head called Muecas for affective HRI is shown in [128]; the robot has 3 DOFs eyeballs, 4 DOFs eyebrows, and 1 DOF mouth; pitch eyeball movements are enabled by Faulhaber LM-2070 linear direct current (DC) servomotor via a linear guide mechanism, while two Faulhaber LM-1247 linear DC servomotors directly provide independent yaw eyeball movements; eyebrows are directly actuated by Hitec HS-45HB servomotors; the robot has a stereo audio system—speakers and microphones, vision system consisting of stereo cameras Point Gray Dragonfly2 IEEE-1394 with custom control sensor (CCS) and controller, as well as red green blue-depth (RGB-D) sensor; the robot is capable of producing 4 facial expressions (sadness, happiness, fear, and anger).

A mobile humanoid robotic platform called Robovie designed for HRI is shown in [129]; the robot has 4 DOFs eyeballs for gaze control actuated by direct-drive motors; also, it has obstacle detection sensors, tactile sensors, omnidirectional vision sensor, and microphones for receiving and recognizing voice commands.

The social robot SyPEHUL (System of Physics, Electronics, HUmanoid robot and machine Learning) is shown in [130]; the robot has 2 DOFs eyeballs, 2 DOFs eyebrows, 4 DOFs mouth, and 2 DOFs ears—all joints are actuated by servo motors, while facial expression recognition camera is located between the eyes; the robot is able to produce 4 facial expressions (happiness, sadness, anger, and surprise).

A huggable social robot called Probo designed for HRI research with a focus on non-verbal communication is shown in [131]; the robot has 3 DOFs eyeballs, 2 DOFs eyelids, 4 DOFs eyebrows, 2 DOFs lips, 2 DOFs ears, and a 1 DOF jaw, where the eyeballs, eyelids, and eyebrows are actuated by compliant Bowden cable-driven actuators (CBCDAs); also, it has a charge-coupled device (CCD) vision camera located between the eyes, sound processing microphones, and force sensor resistors for touch; the robot is capable of producing 6 facial expressions (anger, disgust, fear, happiness, sadness, and surprise).

A humanoid research platform called CB (Computational Brain) for exploring neuroscience is presented in [132]; the robot has 4 DOFs eyeballs and two cameras in each eye—Elmo MN42H 17 mm OD (peripheral) and Elmo QN42H 7 mm OD (foveal) for visual processing and ocular-motor responses using sensors and vision software; in addition, stereo microphones enable the robot’s sense of hearing after perceptual signal processing.

A humanoid head called Amir-II with emotion expression capabilities is shown in [133]; the robot has 2 DOFs eyelids, 2 DOFs eyebrows, and 3 DOFs mouth—all joints are actuated Dynamixel AX-12 servomotors, while a universal serial bus (USB) webcam mounted on the robot’s head is used for vision; the robot is capable of producing 4 facial expressions (happiness, anger, sadness, and disgust).

A humanoid robotic torso called James designed to operate in an unstructured environment is shown in [134,135]; the robot has 4 DOFs eyeballs with two digital CCD Point Gray Dragonfly cameras in them actuated by Faulhaber motors via tendon-driven mechanisms; also, it has Intersense iCube2 3-axis orientational tracker (vestibular system) mounted on the head, while the pressure sensors are used for tactile information.

An infant-like robot called Infanoid, designed to investigate the underlying mechanisms of social intelligence is presented in [136]; the robot has 3 DOFs eyeballs, 2 DOFs eyebrows, and 2 DOFs lips; 2 different color CCD cameras with wide angle and telephoto lens for object recognition and focusing, respectively, are located in each eyeball, while 3 motors actuate them enabling saccade (over 45° within 100 ms) and smooth pursuit movements.

A mobile humanoid robot called Robotinho—a tour guide with multimodal interaction capabilities is shown in [137]; the robot has 4 DOFs eyes represented by 2 USB cameras, 4 DOFs eyebrows, upper eyelids with 1 DOF, while lower eyelids move together with eyeballs, as well as jaw and mouth with a total of 6 FOFs—all joints are actuated by small digital Dynamixel servos; also, it has an attitude sensor (dual-axis accelerometer and two gyroscopes), 8 ultrasonic distance sensors Devantech SRF02 and laser range finder (LRF); the robot is capable of producing 6 facial expressions (surprise, fear, joy, sadness, anger, and disgust).

An emotion-display robot called EDDIE (Emotion Display with Dynamic Intuitive Expressions) is shown in [138]; the robot has 3 DOFs eyeballs, 4 DOFs eyelids, and 4 DOFs eyebrows that are actuated by miniature Atom Mini servomotors via levers and rings, while FireWire cameras are located in the eyeballs; in addition to the vision sensor, two microphones for sound identification and speech recognition are located on the head; the robot is able to produce 6 facial expressions (joy, surprise, anger, disgust, sadness, and fear).

An active vision humanoid head robot called MERTZ is shown in [139]; the robot has 3 DOFs eyeballs, 2 DOFs eyebrows, and 1 DOF for upper eyelids; Point Gray OEM Dragonfly cameras in the eyes are located allowing visual input, while the GN Netcom VA-2000 voice array desk microphone allows interaction with multiple people simultaneously; in addition, the robot has force sensors and motor encoders.

A mobile-dexterous-social robot called MDS Nexi with a highly articulate face for HRI research is shown in [140,141]; the robot has 3 DOFs eyeballs, 2 DOFs eyelids, 2 eyebrows, and 3 DOFs jaw; FireWire color cameras with a 6 mm microlens are located in the eyeballs, while a three-dimensional infrared (3D IR) depth-sensing camera for facial and object recognition is placed on the robot’s forehead, along with 4 microphones to localize the sound; all joints are equipped with current sensors and high-resolution encoders; the robot is capable of producing several facial expressions, such as anger, confusion, excitement, boredom, etc.

Karlsruhe humanoid head—an experimental platform for the realization of interactive service tasks and cognitive vision research is presented in [142]; the robot has 4 DOFs eyeballs that activate Harmonic Drive motors with backlash-free gears and Faulhaber DC motors with backlash-free gears enabling pitch and yaw movements, respectively; two Point Gray Dragonfly2 IEEE-1394 cameras (wide-angle lens for peripheral vision and narrow-angle lens for foveal vision) are located in each eyeball, and the robot has an acoustic sensor (six channel microphone system) and inertial system (encoders, gyroscope).

### 3.2. Hybrid Robot Face—Moving Mechanical Parts and the Use of LEDs

An interactive robotic cat called iCat with both object and facial recognition capabilities is shown in [143,144]; the robot has 3 DOFs eyeballs, 2 DOFs eyelids, 2 DOFs eyebrows, and 4 DOFs mouth—all joints are actuated by radio control (RC) servo motors, while the camera for recognizing objects and faces is located in the nose; also, it has an audio system—microphones for receiving, recording sound signals, recognizing speech and its direction, as well as a speaker for generating speech, tactile sensors, and multi-color LEDs in the ears and legs for more efficient emotion expressions; the robot is capable of producing 6 facial expressions (happiness, surprise, fear, sadness, disgust, and anger).

The Bielefeld anthropomorphic robot head called Flobi with human-like appearance is shown in [145]; the robot has 3 DOFs eyeballs and Point Gray Dragonfly2 in each eye, 4 DOFs eyelids, 2 DOFs eyebrows, and 6 DOFs mouth; eyeballs and eyebrows actuated Maxon motors via levers and tendon-driven mechanisms, respectively; also, it has red green blue (RGB) sensor and M12 micro lenses, high sensitivity microphone, two different gyroscopes, and LEDs in the cheeks that change colors in accordance with the expressed emotion; the maximum angular speed of saccadic movements is 500°/s; the robot is capable of producing 5 facial expressions (happiness, sadness, anger, surprise and fear).

An interactive robot called Golden Horn with emotion expression capabilities and face detection is shown in [146]; the robot has 4 DOFs eyeballs and 4 DOFs upper eyelids actuated by artificial intelligence (AI) motors; in addition, it has LEDs in the cheeks to generate certain emotions, while the webcam and microphone are encapsulated in the eyeballs allowing face detection and voice recognition, respectively; the robot is capable of producing 6 basic and several additional facial expressions (happiness, anger, sadness, surprise, disgust, and fear, as well as sleepiness, innocence, disregard, nervousness, dizziness, and doubtfulness).

A bipedal humanoid robot called Romeo with gaze-shifting capabilities is shown in [147]; the robot has 4 DOFs eyeballs that actuate brushed Maxon DC motors via proximal links; the maximum controllable and non-controllable angular speed of the eyeball is 450°/s and 1000°/s, respectively; the robot has two Aptina Imaging MT9M114 cameras located in the eyeballs, LEDs for displaying the mouth, microphones, and speakers, and tactile sensors and depth sensor for navigation and perception.

An open humanoid platform called Epi designed for experiments in developmental robotics is presented in [148]. What sets the Epi apart from other robots is its eyes with controllable pupils and iris color; the robot has 4 DOFs eyeballs actuated by Dynamixel servomotors enabling yaw eyeball movements and animated pupil movements (the inner body of the eye contains an LED ring and 12 independently controlled RGB diodes), while pitch eyeball movements are not possible; the maximum angular speed of the horizontal saccades is 475°/s; the robot has cameras in both eyes located for stereo vision, contact, and bend sensors in hands, and LEDs for generating lips.

An expressive bear-like robot called eBear for exploration of HRI including verbal and non-verbal communication is shown in [149]. The robot has 2 DOFs eyeballs, 2 DOFs eyelids, 2 DOFs eyebrows, and 2 DOFs ears—all joints are actuated by Hitec PWM servomotors; also, it has a camera to recognize facial expressions with an appropriate visual recognition system and LEDs to display the mouth and generate different emotions; the robot is capable of producing 6 facial expressions (joy, anger, sadness, disgust, surprise, and fear).

An open source humanoid robotic platform called iCub, designed explicitly to support research in embodied cognition is shown in [150]. The robot has 3 DOFs eyeballs—the cameras are located in the eyeballs, and brushed Faulhaber DC motors via toothed belts actuate them, while the eyebrows and mouth are displayed using LEDs allowing basic facial expressions; in addition, the robot has vestibular, auditory, and haptic sensory capabilities.

The Twente humanoid head designed as a research platform for human-machine interaction (HMI) is presented in [151]; the robot has 3 DOFs eyeballs in which CCD cameras are located to track objects and perceive human facial expressions, while the eyebrows and mouths are displayed using LEDs enabling human-like facial expressions.

A multifunctional emotional biped humanoid robot called KIBO with facial expression capabilities and various human-interactive devices is shown in [152]; the robot has 4 DOFs eyeballs, 4 DOFs eyelids, 2 DOFs eyebrows, and 5 DOFs lips; stereo cameras are located in the eyeballs, while the actuation of the joints is performed by small Hitec RC servo motors; in addition, it has a camera for position assessment, microphones for voice recognition, an ultrasonic sensor for front obstacle detection and distance measurement, as well as a lower ground camera for floor obstacle detection; using LEDs, the robot changes color depending on the situational context and the expressed emotion.

Emotion expression humanoid robot called WE-4RII (Waseda Eye No.4 Refined II) is shown in [153]; the robot has 3 DOFs eyeballs in which CCD cameras are located, 6 DOFs eyelids, 8 DOFs eyebrows, 4 DOFs lips, and a 1 DOF jaw; pitch eyeball movements are enabled by a DC motor and harmonic drive system via a belt-driven mechanism, while independent yaw eyeball movements are enabled by DC motors and torsion springs via tendon-driven mechanisms—the eyelids are actuated in a similar way; the maximum angular speed of the eyeball is 600°/s, while one blink lasts 0.3 s and achieves a speed of 900°/s, which is similar to a human; the robot has microphones, temperature sensors, tactile sensors, gas sensors, and force sensors, while the cheeks change color in accordance with the expressed emotion using electroluminescence (EL); in addition to speech recognition, the robot is capable of producing 6 facial expressions (happiness, surprise, anger, disgust, fear, and sadness).

### 3.3. Summary

Based on a review of the available literature and analysis of the results, we conclude: (i) robots typically have 3 or 4 DOFs eyeballs allowing common pitch and independent yaw movements or independent pitch and yaw movements of each eye, respectively; (ii) the robots generally have 2 or 4 DOFs eyelids allowing the upper eyelids to rotate independently (while the lower eyelids are stationary or move in accordance with the vertical saccades of the eye) or each eyelid to move independently, respectively; (iii) robots typically have 2 or 4 DOFs eyebrows allowing independent rotation or translation of the eyebrows, or independent rotation and translation of each eyebrow, respectively; (iv) the transmission of motion from actuators to eyeballs, eyelids, and eyebrows is typically realized using gears, levers and rings, tendon-driven mechanisms, belt-driven mechanisms, cable-driven mechanisms, linear-guide mechanisms or direct-drive actuators; (iv) joint actuation is most commonly performed by servomotors, while Maxon and Faulhaber DC motors are less commonly used; (v) cameras can be located in the eyeballs—one or two in each eye allowing perception of the environment, recognition of faces and objects using vision and image processing systems, however, most robots have cameras located on the head, forehead, nose or chest; (vi) robots generally have one or more microphones for receiving and processing audio signals, as well as a speaker for transmitting verbal messages; (vii) in the end, only one robot has developed eyes and eyebrows in accordance with the biological and kinematic principles of the human eye.

## 4. Problem Description

**Figure 3:**
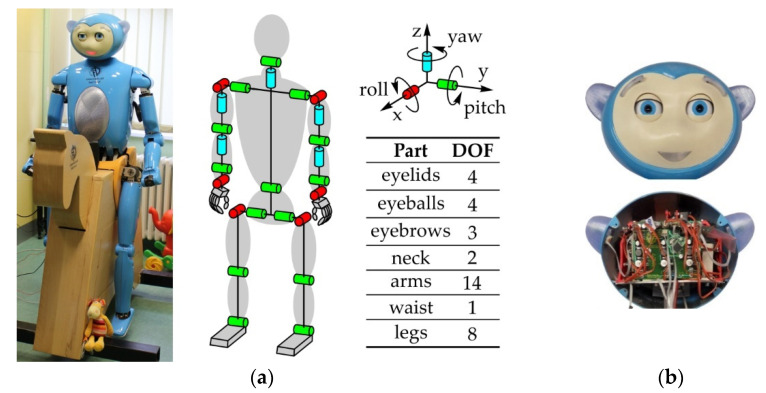
(**a**) Human-like robot MARKO and its kinematic structure; (**b**) Robot head.

During the previous two decades, robotics have developed a presence within the field of healthcare, and its technologies are generally accepted by doctors, nurses, and patients [154,155,156]. The use of SARs in therapy for people with autism spectrum disorder (ASD) [157,158,159], cerebral palsy (CP) [160,161,162], and dementia [163,164,165] has had positive effects. Additionally, the use of robots as emotional and social support for persons with mild cognitive impairment or older people who are alone and/or lonely, has been the subject of many studies [166,167,168,169]. Figure 3a shows the human-like robot MARKO, which is used as a motivational tool in physical therapy for children with CP [170]. Due to the nature of CP and that no two children will have identical clinical manifestations, it is key to discover the illness within the first few years of life, determine the diagnosis, and begin physical therapy, which is the cornerstone of CP treatment [171,172,173]. One of the goals of physical therapy is to strengthen the musculature and improve the fine motor skills of the child, with success being directly dependent on how willing the child is to do the exercises thus preventing contractures. However, although the successfulness of the therapy is directly proportional to its duration, the problem with executing these exercises is due to brain damage—the movements are often strenuous, painful, and tiring, so the child will very quickly lose interest in working with the therapist.

According to the clinical study shown in [170], it was determined that the robot MARKO raises the interest of the children to complete the exercises, motivates, and encourages them to exercise longer when compared to the conventional approach, thus making the therapy more successful. The robot firstly engages the child verbally, after which it begins demonstrating the exercise. The child then must repeat the exercise as many times as they can. After every completed exercise, the robot rewards the child with praise. It was noted that every child needs a script tailored to them specifically and that children, in general, perceive the robots as human beings. Due to this, the robot should be able to express emotions in a human-like way, in line with the kinematic principles of the eye and eyebrows. The robot has 4 DOFs eyeballs, 4 DOFs eyelids, and 3 DOFs eyebrows, as well as LEDs for the mouth and ears. CCD Fire-i board cameras are located within the eyeballs, while all the joints are actuated using Modelcraft servos. Additionally, it has a microphone, a speaker, and a system for speech recognition and synthesis.

The mechanical systems of the eyeballs, eyelids, and eyebrows are the subject being reconstructed in this paper for a number of reasons: (i) the eyes and eyebrows of the robot are not capable of producing the types of motions, the ranges of motion or the speeds of their human counterparts, and having these capabilities would, on a functional level, enable the spectrum of movements necessary for the simulation of emotional expressions, which is a key feature; (ii) the existing actuators are not capable of producing speeds appropriate for human-like motion, with the output link having high values of arc backlash, which negatively impacts the positioning accuracy and the repeatability of the output link motion, especially since the cameras are located in the eyeballs; also, this problem causes jerks when movements are initiated, which negatively impacts the stability of the picture; (iii) the dimensions and shape of the actuators directly influenced the structure of the mechanical system and the mechanism dimensions—the structure is not optimized, and the dimensions are too large, making the eye modules take up most of the head’s volume (see Figure 3b); a consequence of this are potential problems during motion—unfavorable transmission angles and low mechanical advantage would cause most of the power to be wasted on overcoming internal friction in the mechanism joints; (iv) the driving mechanisms of the eyes are linkage mechanisms, but the links are imprecisely bent which caused issues with the kinematics; (v) the eye module dimensions directly impacted the structure and dimensions of the eyebrow rotation and translation mechanism; due to this, the eyebrows were positioned outside of the eye region (see Figure 3b) which is not in line with the anthropometrics of the face; in effect, the eyebrows are not functional due to the driving mechanism being inadequate because of the lack of space in the head; (v) each eyeball has 2 DOFs and due to everything stated so far, there are inconsistencies when realizing the saccades which manifests with strabismus; an additional problem is the realization of vergence movements for focusing objects in the line of sight; (vi) the base platforms of the eyes and eyebrows were made using 3D binder jetting technology and 3D printing technology, respectively; the consequences of this are manufacturing errors due to deformation during the hardening and cooling of the material, which has negative effects on the positioning accuracy and part assembly; all of this caused further problems, such as backlash in the joints and high values of friction.

### Summary

The goal of this paper is the structural design of a new mechanical and control system of robot eyes, which will functionally enable an assortment of movements that human eyes and eyebrows are capable of, to simulate the emotional state of the robot. The mechanical system must represent an adequate hardware platform for the development and implementation of robotic vision and algorithms with different purposes, such as face and object detection, emotion recognition, semantic segmentation of scenes, etc. By using a vision system, supported by a sophisticated mechanical and control system, robots could lower the burden carried by the healthcare system, contributing to the quality of care of ill and threatened persons, as well as to the safety of healthcare workers.

## 5. Mechanical System

The mechanical system shown in this paper consists of three independent subassemblies: (i) the mechanical system of the eyeballs, (ii) the mechanical system of the eyelids, and (iii) the mechanical system of the eyebrows. Due to their independence, each of them will be considered with regard to their structure and preliminary dimensions.

### 5.1. Structural Design

The following text presents the structure of the systems driving the eyeballs, eyelids, and eyebrows, as well as the basic equations describing their kinematic behavior.

#### 5.1.1. Mechanical System of the Eyeballs

**Figure 4:**
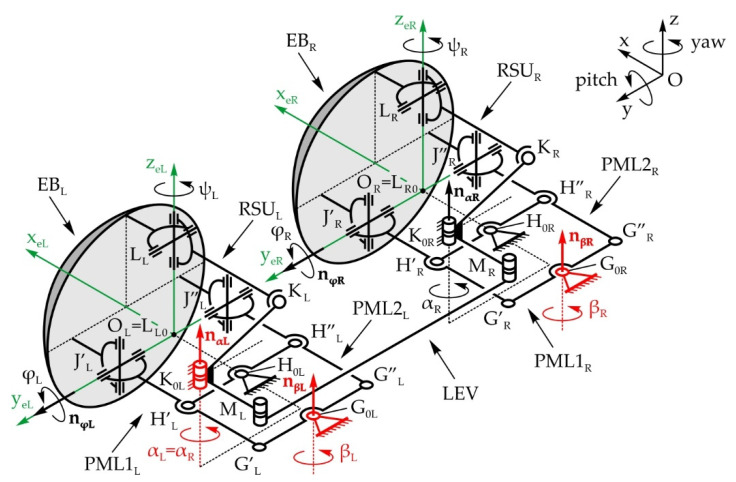
Eyeballs’ mechanical system with total 3 DOFs. Note: the indexes L and R refer to the left and right eyeball, respectively.

Figure 4 shows the structure of the eyeball mechanical system with a total of 3 DOFs, allowing the pitch and yaw motions of the eyeball—angles φ_L/R_ and ψ_L/R_, respectively. The mobile platforms marked as L_L/R_, J’_L/R_, and J″_L/R_ are the eyeballs, realized as spheres with center points in O_L/R_. The base platforms are integrated with the robot head frame, and are defined with points K_0(L/R)_, H_0(L/R)_, and G_0(L/R)_. The motion of the eyeball is defined with one RSU_L/R_ leg (R, S, and U stand for revolute, spherical, and universal joints, respectively) and two identical PML1_L/R_ and PML2_L/R_ legs which form planar four-bar linkages with parallelogram configurations G_0(L/R)_, G’_L/R_, H’_L/R_, H_0(L/R)_ and G_0(L/R)_, G″_0(L/R)_, H″_L/R_, H_0(L/R)_, respectively. The RSU_L/R_ leg provides the pitch rotation—angle φ_L/R_, while the PML1_L/R_ and PML2_L/R_ legs provide the yaw rotation to the eyeball—angle ψ_L/R_. The motion is achieved with 3 actuators placed in joints K_0L_, G_0L_, and G_0R_. However, the PML1_L/R_ and PML2_L/R_ legs are driven by the same actuator, since levers G_0(L/R)_, G’_(L/R)_ and G_0(L/R)_, G”_0(L/R)_ are fixed to one another. The four-bar linkage marked as LEV transmits the motion from the actuator in joint K_0L_ to passive joint K_0R_, therefore making α_L_ = α_R_. The axes’ unit vectors of the R joints are marked as **n_α(L/R)_** and **n_φ(L/R)_**. Due to the joint structure, the eyeball can complete pitch and yaw motions, either independently or simultaneously. The eyeball center does not move during either motion, making the motion of the eyeball spherical with regard to its center. The local coordinate system O_L/R_x_e(L/R)_y_e(L/R)_z_e(L/R)_ is fixed to the eyeball and in the initial position, the directions of the axes coincide with the axes of the fixed global coordinate system Oxyz. Since the mechanisms of both the left and right eyeball are structurally identical, the indexes denoting left L and right R will be omitted in the following text.

According to the input parameters of the eyeball driving system: the lever lengths and the position angles of the mechanism input links—angles α and β—the following output kinematic parameters are determined: the position—angles φ and ψ, and the angular velocities of the eyeball. Firstly, the pitch motion of the eyeball is considered, defined by the rotation angle φ:

(1) $$\varphi =2\arctan\left(\frac{-a+\sqrt{a^{2}+b^{2}-c^{2}}}{c-b}\right)$$

where:

(2) $$a={\left(k-l_{0}\right)}^{T}\left[P_{n\alpha }\right]\left(l_{s}-l_{0}\right)$$

(3) $$b={\left(k-l_{0}\right)}^{T}\left[I-Q_{n\alpha }\right]\left(l_{s}-l_{0}\right)$$

(4) $$c={\left(k-l_{0}\right)}^{T}\left[Q_{n\alpha }\right]\left(l_{s}-l_{0}\right)+\frac{1}{2}{\left(k_{s}-l_{s}\right)}^{T}\left(k_{s}-l_{s}\right)-\frac{1}{2}{\left(k-l_{0}\right)}^{T}\left(k-l_{0}\right)-\frac{1}{2}{\left(l_{s}-l_{0}\right)}^{T}\left(l_{s}-l_{0}\right)$$

(5) $$k=\left[R_{\alpha ,n\alpha }\right]\left(k_{s}-k_{0}\right)+k_{0}$$

(6) $$l=\left[R_{\varphi ,n\varphi }\right]\left(l_{s}-l_{0}\right)+l_{0}$$

where:

**k** and **l**—position vectors of points K and L,
**k_0_** and **l_0_**—position vectors of immobile points K_0_ and L_0_,
**k_s_** and **l_s_**—position vectors of points K and L in initial position,
[**R_α_**_,**nα**_] and [**R_φ_**_,**nφ**_]—rotation matrix, and
[**P_nα_**] and [**Q_nα_**]—corresponding matrixes.

Rotation matrix [**R_α,nα_**], rotation α around an axis **n_α_** = (n_ax_,n_ay_,n_az_) is determined according to:

(7) $$\left[R_{\alpha ,n\alpha }\right]=\left[\begin{array}{ccc} n_{\alpha x}^{2}\left(1-\cos\alpha \right)+\cos\alpha & n_{\alpha x}n_{\alpha y}\left(1-\cos\alpha \right)-n_{\alpha z}\sin\alpha & n_{\alpha x}n_{\alpha y}\left(1-\cos\alpha \right)+n_{\alpha z}\sin\alpha \\ n_{\alpha x}n_{\alpha y}\left(1-\cos\alpha \right)+n_{\alpha z}\sin\alpha & n_{\alpha y}^{2}\left(1-\cos\alpha \right)+\cos\alpha & n_{\alpha y}n_{\alpha z}\left(1-\cos\alpha \right)-n_{\alpha x}\sin\alpha \\ n_{\alpha x}n_{\alpha z}\left(1-\cos\alpha \right)-n_{\alpha y}\sin\alpha & n_{\alpha y}n_{\alpha z}\left(1-\cos\alpha \right)+n_{\alpha x}\sin\alpha & n_{\alpha z}^{2}\left(1-\cos\alpha \right)+\cos\alpha \end{array}\right]$$

Rotation matrix [**R_φ,nφ_**], rotation φ around an axis **n_φ_** = (n_φx_,n_φy_,n_φz_) is determined according to:

(8) $$\left[R_{\varphi ,n\varphi }\right]=\left[\begin{array}{ccc} n_{\varphi x}^{2}\left(1-\cos\varphi \right)+\cos\varphi & n_{\varphi x}n_{\varphi y}\left(1-\cos\varphi \right)-n_{\varphi z}\sin\varphi & n_{\varphi x}n_{\varphi z}\left(1-\cos\varphi \right)+n_{\varphi y}\sin\varphi \\ n_{\varphi x}n_{\varphi y}\left(1-\cos\varphi \right)+n_{\varphi z}\sin\varphi & n_{\varphi y}^{2}\left(1-\cos\varphi \right)+\cos\varphi & n_{\varphi y}n_{\varphi z}\left(1-\cos\varphi \right)-n_{\varphi x}\sin\varphi \\ n_{\varphi x}n_{\varphi z}\left(1-\cos\varphi \right)-n_{\varphi y}\sin\varphi & n_{\varphi y}n_{\varphi z}\left(1-\cos\varphi \right)+n_{\varphi x}\sin\varphi & n_{\varphi z}^{2}\left(1-\cos\varphi \right)+\cos\varphi \end{array}\right]$$

Μatrix [**P_nα_**] and [**Q_nα_**] are determined according to:

(9) $$\left[P_{n\alpha }\right]=\left[\begin{array}{ccc} 0 & -n_{\alpha z} & n_{\alpha y}\\ n_{\alpha z} & 0 & -n_{\alpha x}\\ -n_{\alpha y} & n_{\alpha x} & 0 \end{array}\right]$$

(10) $$\left[Q_{n\alpha }\right]=\left[\begin{array}{ccc} n_{\alpha x}^{2} & n_{\alpha x}n_{\alpha y} & n_{\alpha x}n_{\alpha z}\\ n_{\alpha x}n_{\alpha y} & n_{\alpha y}^{2} & n_{\alpha y}n_{\alpha z}\\ n_{\alpha x}n_{\alpha z} & n_{\alpha y}n_{\alpha z} & n_{\alpha z}^{2} \end{array}\right]$$

The angular speed of output link LL_0_ is:

(11) $$\dot{\varphi }=\frac{{\left(\dot{k}\right)}^{T}\left(k-l\right)}{{\left(k-l\right)}^{T}\left[P_{n\varphi }\right]\left(l-l_{0}\right)}$$

The velocity of point K is known and equals:

(12) $$\dot{k}=\;\dot{\alpha }\left[P_{n\alpha }\right]\left(k-k_{0}\right)$$

Now the velocity of point L on the eyeball is determined:

(13) $$\dot{l}=\;\dot{\varphi }\left[P_{n\varphi }\right]\left(l-l_{0}\right)$$

The yaw motion of the eyeball is considered next. The positions of points J’ and J” are defined by vectors **j’** and **j”**, respectively (x_O_ and y_O_ are the coordinates of the eyeball center):

(14) $$j^{\prime }=\left(-\bar{{\;G\prime G}_{0}}\sin\beta +x_{O},\bar{{\;G\prime G}_{0}}\cos\beta +y_{O},0\right)$$

(15) $$j^{\prime \prime }=\left(\bar{{\;G\prime \prime G}_{0}}\sin\beta +x_{O},-\bar{{\;G\prime \prime G}_{0}}\cos\beta +y_{O},0\right)$$

Since G_0_G’ = G_0_G”, the eyeball rotates about the z-axis when angle β changes. Due to this, the position of the eyeball—angle ψ, is equal to the position of the input link—angle β, therefore:

(16) $$\psi =\beta ,\;\dot{\psi }=\;\dot{\beta }$$

#### 5.1.2. Mechanical System of the Eyelids

**Figure 5:**
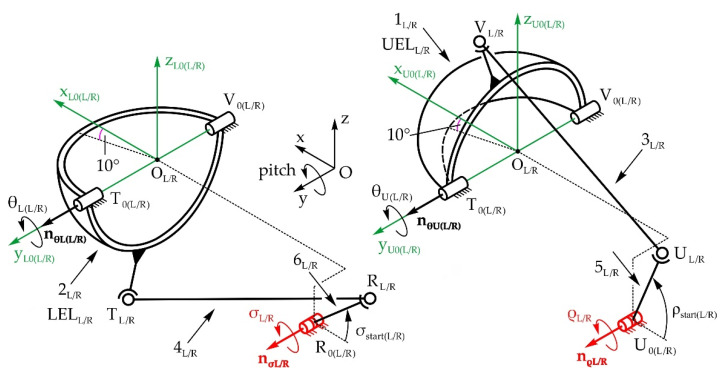
Eyelids’ mechanical system with total 4 DOFs. Note: the indexes L and R refer to the left and right eye, respectively.

Figure 5 shows the structure of the eyelid mechanical system with a total of 4 DOFs, which enables the rotation of the upper and lower eyelids—angles θ_U(L/R)_ and θ_L(L/R)_, respectively. The upper/lower eyelids UEL_L/R_ and LEL_L/R_ are spherical shells with centers in points O_L/R_ (the eyeball center points). The mechanical system consists of four spatial mechanisms with RSSR configurations, driven by actuators placed in joints U_0(L/R)_ and R_0(L/R)_. The unit vectors of the R joint axes are **n_θU(L/R)_** and **n_ρL/R_** for the upper eyelid, and **n_θL(L/R)_** and **n_σL/R_** for the lower eyelid. The local coordinate systems are fixed to the appropriate eyelid and in the initial position, the directions of the axes coincide with the fixed global coordinate system Oxyz. The eyelids are open in the initial position. When they close, the plane where they make contact lies along the y-axis and is at a 10° angle relative to the horizontal plane. Since the mechanisms of both the left and right eyelid are structurally identical, the indexes denoting left L and right R will be omitted in the following text.

Based on the input kinematic parameters of the eyelid driving system: the lever lengths and the positions of the input links—angles ρ and σ—the output kinematic parameters are defined: the positions—angles θ_U_ and θ_L_, and angular velocities of the eyelids. Firstly, the position of the upper eyelid is determined:

(17) $$\theta_{U}=2\arctan\left(\frac{-a+\sqrt{a^{2}+b^{2}-c^{2}}}{c-b}\right)$$

where:

(18) $$a={\left(u-v_{0}\right)}^{T}\left[P_{n\theta U}\right]\left(v_{s}-v_{0}\right)$$

(19) $$b={\left(u-v_{0}\right)}^{T}\left[I-Q_{n\theta U}\right]\left(v_{s}-v_{0}\right)$$

(20) $$c={\left(u-v_{0}\right)}^{T}\left[Q_{n\theta U}\right]\left(v_{s}-v_{0}\right)+\frac{1}{2}{\left(u_{s}-v_{s}\right)}^{T}\left(u_{s}-v_{s}\right)-\frac{1}{2}{\left(u-v_{0}\right)}^{T}\left(u-v_{0}\right)-\frac{1}{2}{\left(v_{s}-v_{0}\right)}^{T}\left(v_{s}-v_{0}\right)$$

(21) $$u=\left[R_{\rho ,n\rho }\right]\left(u_{s}-u_{0}\right)+u_{0}$$

(22) $$v=\left[R_{\theta ,n\theta U}\right]\left(v_{s}-v_{0}\right)+v_{0}$$

where:

**u** and **v**—position vectors of points U and V,
**u_0_** and **v_0_**—position vectors of immobile points U_0_ and V_0_,
**u_s_** and **v_s_**—position vectors of points U and V in initial position,
[**R_ρ_**_,**n**_**_ρ_**] and [**R_θ_**_,**n**_**_θU_**]—rotation matrix (see Equations (7) and (8), respectively), and
[**P_nθU_**] and [**Q_nθU_**]—corresponding matrixes (see Equations (9) and (10), respectively).

The angular speed of output link VV_0_ is:

(23) $${\dot{\theta }}_{U}=\frac{{\left(\dot{u}\right)}^{T}\left(u-v\right)}{{\left(u-v\right)}^{T}\left[P_{n\theta U}\right]\left(v-v_{0}\right)}$$

The velocity of point U is known and equals:

(24) $$\dot{u}=\;\dot{\rho }\left[P_{n𝟈}\right]\left(u-u_{0}\right)$$

Now the velocity of point V on the upper eyelid is determined as:

(25) $$\dot{v}={\;\dot{\theta }}_{U}\left[P_{n\theta U}\right]\left(v-v_{0}\right)$$

The position of the lower eyelid is determined as:

(26) $$\theta_{L}=2\arctan\left(\frac{-a+\sqrt{a^{2}+b^{2}-c^{2}}}{c-b}\right)$$

where:

(27) $$a={\left(r-t_{0}\right)}^{T}\left[P_{n\theta L}\right]\left(t_{s}-t_{0}\right)$$

(28) $$b={\left(r-t_{0}\right)}^{T}\left[I-Q_{n\theta L}\right]\left(t_{s}-t_{0}\right)$$

(29) $$c={\left(r-t_{0}\right)}^{T}\left[Q_{n\theta L}\right]\left(t_{s}-t_{0}\right)+\frac{1}{2}{\left(r_{s}-t_{s}\right)}^{T}\left(r_{s}-t_{s}\right)-\frac{1}{2}{\left(r-t_{0}\right)}^{T}\left(r-t_{0}\right)-\frac{1}{2}{\left(t_{s}-t_{0}\right)}^{T}\left(t_{s}-t_{0}\right)$$

(30) $$r=\left[R_{\sigma ,n\sigma }\right]\left(r_{s}-r_{0}\right)+r_{0}$$

(31) $$t=\left[R_{\theta ,n\theta L}\right]\left(t_{s}-t_{0}\right)+t_{0}$$

where:

**r** and **t**—position vectors of points R and T,
**r_0_** and **t_0_**—position vectors of immobile points R_0_ and T_0_,
**r_s_** and **t_s_**—position vectors of points R and T in initial position,
[**R_σ,nσ_**] and [**R_θ,nθL_**]—rotation matrix (see Equations (7) and (8), respectively), and
[**P_nθL_**] and [**Q_nθL_**]—corresponding matrixes (see Equations (9) and (10), respectively).

The angular speed of link TT_0_ equals:

(32) $${\dot{\theta }}_{L}=\frac{{\left(\dot{r}\right)}^{T}\left(r-t\right)}{{\left(r-t\right)}^{T}\left[P_{n\theta L}\right]\left(t-t_{0}\right)}$$

The velocity of point R is known and equals:

(33) $$\dot{r}=\;\dot{\sigma }\left[P_{n\sigma }\right]\left(r-r_{0}\right)$$

Now the velocity of point T on the lower eyelid is determined as:

(34) $$\dot{t}={\dot{\theta }}_{L}\left[P_{n\theta L}\right]\left(t-t_{0}\right)$$

#### 5.1.3. Mechanical System of the Eyebrows

**Figure 6:**
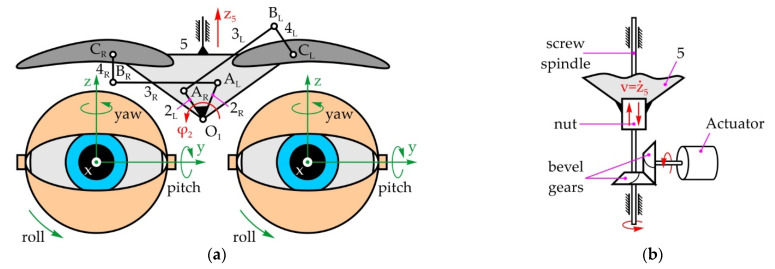
(**a**) Eyebrows’ mechanical system with total 2 DOFs; (**b**) Spindle drive mechanism.

Figure 6a shows the mechanical system of the eyebrows with a total of 2 DOFs, enabling rotational and translational motion of the eyebrows—angle φ_2_ and displacement z_5_ along the vertical axis, respectively. The eyebrows’ rotation mechanism consists of two levers, 2_L_ and 2_R_, which are fixed to each other, becoming input link 2, levers 3_L_ and 3_R_—floating links, and levers 4_L_ and 4_R_ which are fixed to the left and right eyebrow, respectively—output links. The eyebrows are raised by link 5 which performs translational motion in relation to the immobile link 1. As shown on Figure 6b, link 5 is fixed to a screw nut which moves along the threaded shaft of a spindle drive mechanism, enabling the transformation of rotational into translational motion. The actuator is position parallel to the x-axis, between the left and right eye modules by way of bevel gears (i = 1).

**Figure 7:**
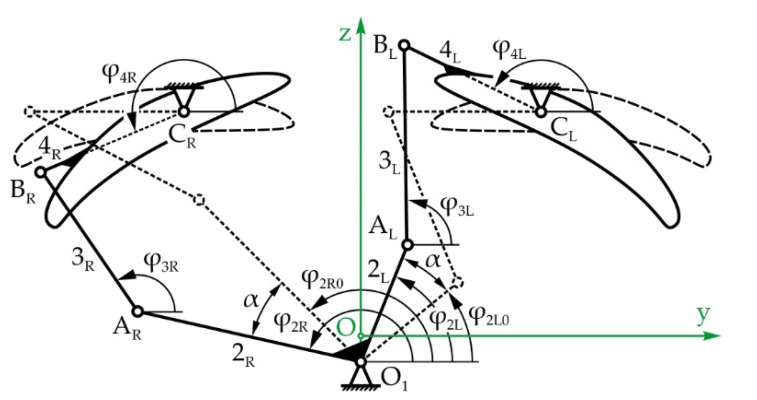
Upper/lower eyelid mechanism.

Figure 7 shows the eyebrow rotation mechanism in its initial—horizontal and rotated positions. During eyebrow rotation, link 5 does not move, so the whole mechanism can be regarded as two independent four-bar linkages.

The lengths of links 2, 3, and 4 for the left and right mechanism are r_2(L/R)_, r_3(L/R)_, and r_4(L/R)_, respectively. The relationship between the eyebrow rotation angle φ_4L/R_ and the input link angle φ_2L/R_ is expressed:

(35) $$\varphi_{4\left(L/R\right)}=\varphi_{d\left(L/R\right)}+\arccos\frac{r_{3\left(L/R\right)}^{2}-d_{L/R}^{2}-r_{4\left(R/L\right)}^{2}}{{2d}_{L/R}r_{4\left(L/R\right)}}$$

where:

(36) $$\varphi_{d\left(L/R\right)}=\arctan\frac{z_{C\left(L/R\right)}-z_{A\left(L/R\right)}}{y_{C\left(L/R\right)}-y_{A\left(L/R\right)}}$$

(37) $$d_{L/R}=\sqrt{{\left(x_{C\left(L/R\right)}-x_{A\left(L/R\right)}\right)}^{2}+{\left(y_{C\left(L/R\right)}-y_{A\left(L/R\right)}\right)}^{2}}$$

where y_C(L/R)_ and z_C(L/R)_ are the coordinates of point C for the left/right mechanism.

The coordinates of point A for the left/right mechanism are:

(38) $$y_{A\left(L/R\right)}=y_{O1}+r_{2\left(L/R\right)}{\cos\varphi }_{2\left(L/R\right)}=y_{O1}+r_{2\left(L/R\right)}\cos\left(\varphi_{2\left(L0/R0\right)}+\alpha \right)$$

(39) $$z_{A\left(L/R\right)}=z_{O1}+r_{2\left(L/R\right)}\sin\varphi_{2\left(L/R\right)}=z_{O1}+r_{2\left(L/R\right)}\sin\left(\varphi_{2\left(L0/R0\right)}+\alpha \right)$$

where φ_2(L/R)_ is the position angle of link 2 for the left/right mechanism, φ_2(L0/R0)_ is the input link angle in the initial position where the left/right eyebrow is horizontal, and α is the rotation angle of link 2 with regard to the initial position.

### 5.2. Dimensional Synthesis

The main function of the mechanism is to transmit motion from the input link to the output link. In order to fulfil the aforementioned, it is necessary for the driving force to be efficiently transmitted to the output link—the measure of this efficiency is the transmission index (TI) [174], the value of which depends on the dimensions and current position of the mechanism. When the mechanism moves, the TI value changes within the interval from 0 to 1, with values closer to 1 indicating higher efficiency. Due to this, the dimensional synthesis will be conducted so that the eyeball, eyelid, and eyebrow mechanisms achieve their prescribed ranges of motion while keeping the TI as high as possible.

#### 5.2.1. Mechanisms of Eyeballs 

**Figure 8:**
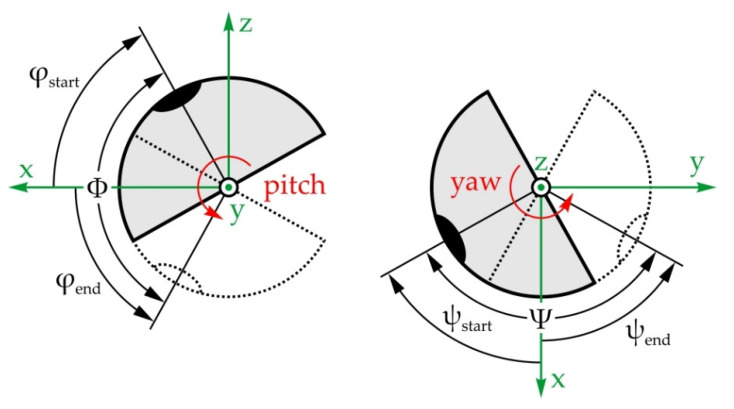
Vertical and horizontal saccadic movements of the eye—pitch and yaw movements.

Figure 8 shows the vertical and horizontal saccadic movements of the eyeball—angle φ and angle ψ, respectively. For the vertical saccadic movements, in the initial position, the eyeball is rotated for φ_start_ = −30° around the y-axis, and then it rotates for the angle Φ = 75° to the end position φ_end_ = 45°. As for the horizontal saccadic movements, in the initial position, the eyeball is rotated for φ_start_ = −45° around the z-axis, and then it rotates for the angle Ψ = 90° to the end position ψ_end_ = 45°. The duration of both movements has been adopted to equal no more than 0.2 s.

In the case of pitch rotation, the eyeball mechanism TI is defined as the cosine of the angle between the direction of the floating link KL and the direction of the velocity of point L [175], therefore:

(40) $${\mathrm{TI}}_{\mathrm{EB}}=\frac{\left|\vec{\mathrm{KL}}\cdot {\;\vec{v}}_{L}\right|}{\left|\vec{\mathrm{KL}}\right|\left|{\;\vec{v}}_{L}\right|}$$

Aside from the prescribed eyeball range of motion and keeping the TI as close to 1 as possible, an additional requirement is the minimization of the mechanism dimensions. Since some of the requirements oppose each other, the dimensional synthesis problem is defined as an optimization problem—minimization of the objective function F(x), x∈D for the set constraints, where x = (x_1_, x_2_,…, x_m_) is the vector of variables, D = {x∈R_n_| g(x) ≤ 0 ˄ h(x) = 0} is the set of solutions that fulfils the defined constraints, while g(x) ≤ 0 and h(x) = 0 are the vectors of constraints. The optimization variables are the geometric parameters of the mechanism: the length of the input link K_0_K, the length of the output link OL, the initial position angle of the input link α_start_, and the range of motion of the input link defined by angle A = |α_end_ − α_start_|. The objective function is therefore formed as:

(41) $$f\left(x\right)=\frac{1}{\left|\mathrm{mean}\;\mathrm{value}\;\mathrm{of}\;{(\mathrm{TI}}_{\mathrm{EB}}{}_{i})\right|}$$

where: TI_EBi_, i = 1,…, n, an array of TI values during eyeball movement.

The desired interval of motion for the eyeball is prescribed, so the following equality constraint is given h_1_ = |φ_end_ − φ_start_| − 75° = 0. The dimensions of the mechanism must be as small as possible, due to the limited space inside the head of the robot, which is also why inequality constraints are introduced (see Table 1).

The following variables are prescribed according to the design requirements—the eyeball center is adopted as the coordinate system origin O(0,0,0). The rotation axis of the input link is parallel to the z-axis, making **n_α_** = (0,0,1); the rotation axis of the output link is parallel to the y-axis, making **n_φ_** = (0,0,1); the position of fixed point K_0_ (position of the actuator) is K_0_ (−80,10,10); in the initial position, point L coincides with the vertical xOz plane, while line OL is at an angle of 120° relative to the x-axis, making $l_{s}=\left(\bar{\mathrm{OL}}\cos120{}^{\circ},0,\bar{\mathrm{OL}}\sin60{}^{\circ}\right).$

According to the previous statements, optimal dimensional synthesis of the RSU leg was conducted, yielding the values shown in Table 2.

**Figure 9:**
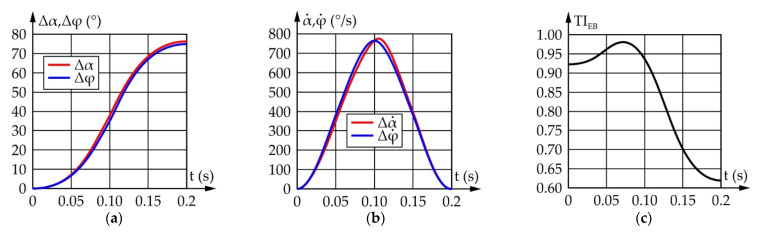
Eyeball motion about the y-axis: (**a**) Angular displacement of the input and output link; (**b**) Angular speed of the input and output link; (**c**) TI for the up-and-down movements.

Figure 9 shows the results of a motion simulation conducted according to the data from Table 2. It should be noted that Δα and Δφ represent the motion of the input and output links relative to their initial positions defined as α_start_ and φ_start_, respectively.

According to Figure 9, to achieve an eyeball range of motion of 75°, the actuator needs to rotate by 76.2°. During which, the maximum angular speed of the eyeball equals 769.1°/s, while the required angular speed of the actuator equals 770.4°/s. The TI value ranges from 0.62 to 0.98, which is satisfactory.

In the case of the yaw motion, the motion is achieved by planar four-bar linkages with parallelogram configurations, meaning the motion of the eyeball is identical to the motion of the actuator (ψ = β) and does not depend on the dimensions of the mechanism. For planar mechanisms, the TI is equivalent to the transmission γ, the angle between the link directions OJ’, H’J’ and OJ”, H”J”, respectively. According to Ref. [176], for lever mechanisms, the recommended bounds for the value of the transmission angle are γ_min_ ≥ 45° and γ_max_ ≤ 135°. In this case, the transmission angle depends solely on the position angle of the input link G_0_G’ and G_0_G”.

**Figure 10:**
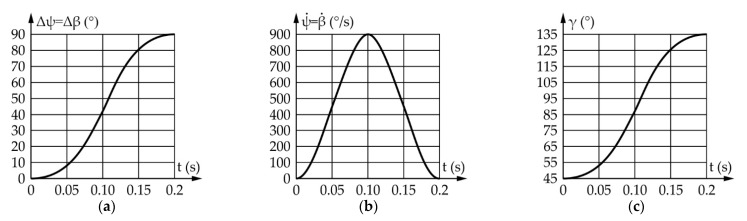
Eyeball motion around the z-axis: (**a**) Angular displacement of input and output link; (**b**) Angular speed of input and output link; (**c**) Transmission angle for the left-and-right movement.

According to everything stated above, a motion simulation was conducted, yielding the results shown in Figure 10. It should be noted that angles Δβ and Δψ represent the motion of the input and output links relative to their initial positions defined as β_start_ and ψ_start_, respectively.

According to Figure 10, the ranges of motion and the angular speeds of the eyeball and actuator are the same and equal, 90° and 769.1°/s, respectively, while the transmission angle value ranges from 45° to 145°, which is satisfactory.

#### 5.2.2. Mechanisms of Eyelids 

**Figure 11:**
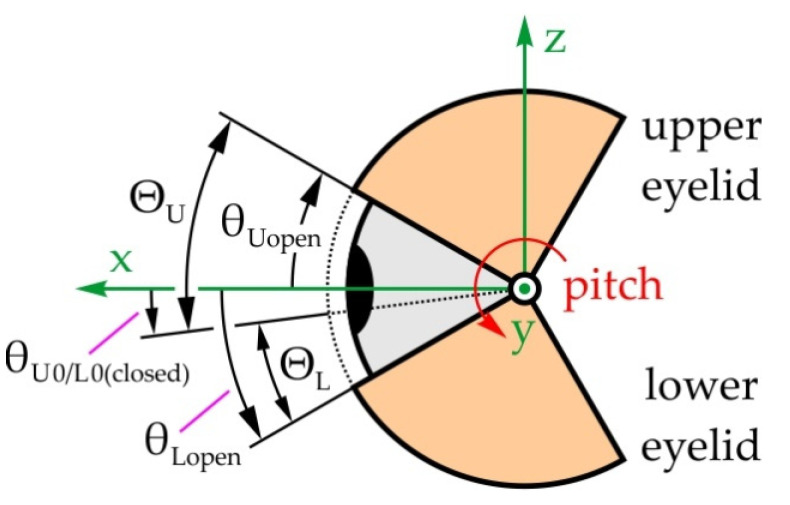
Upper and lower eyelid motion independent to the eyeball.

Figure 11 presents the movement of the eyelids. The ability to adjust how open the eyelids are would enable the generation of a number of emotions, while the ability to blink would make interactions with the robot feel more natural. Due to this, the range of motion and duration of a single blink were defined. In the initial position, the eyelids are position so θ_L0/U0_ = θ_L/U(open)_ = 35°/−40° (please see Figure 11). Then the upper eyelid is rotated for the angle Θ_U_ = 50° to the upper closed position θ_Uclosed_, and the lower eyelid for the angle Θ_L_ = 25° to the lower closed position θ_Lclosed_. In the closed position, the eyelids make contact in a plane angled so that θ_L0/U0_ = θ_L/U(closed)_ = 10°. The eyelids then return to their initial positions. The duration of a single blink was adopted and equals no more than 0.25 s.

The dimensional synthesis will be conducted so the eyelid mechanism achieves the prescribed ranges of motion Θ_U/L_, while keeping the force transmission as favorable as possible. For the upper eyelid mechanism, the TI is defined as the cosine between the direction of the floating link 3 and the direction of the velocity of joint V, meaning:

(42) $${\mathrm{TI}}_{U}=\frac{\left|\vec{\mathrm{UV}}\cdot {\;\vec{v}}_{V}\right|}{\left|\vec{\mathrm{UV}}\right|\left|{\;\vec{v}}_{V}\right|}$$

The TI of the lower eyelid is defined similarly as:

(43) $${\mathrm{TI}}_{L}=\frac{\left|\vec{\mathrm{RT}}\cdot {\;\vec{v}}_{T}\right|}{\left|\vec{\mathrm{RT}}\right|\left|{\;\vec{v}}_{T}\right|}$$

The objective function is therefore formed as:

(44) $$F\left(x\right)=\frac{1}{\left|\mathrm{mean}\;\mathrm{value}\;\mathrm{of}\;{(\mathrm{TI}}_{U/\mathrm{Li}})\right|}$$

where TI_U/Li_, i = 1,…,n, an array of transmission index values during the eyelid motion.

For constraints, the desired interval of motion for the upper and lower eyelid is h_1_ = |θ_Uopen_ − θ_Uclosed_| − 50° = 0 and h_2_ = |θ_Lclosed_ − θ_Lopen_| − 25° = 0, respectively. Additionally, TI_U/L_ should not fall below some acceptable value (set to 0.5), i.e., c_1_ = 0.5 − min *value of* (TI_U/Li_). 

The optimization variables of the upper eyelid are: the length of the input lever U_0_U, the length of the output lever OV, the angle of the input link in the initial position ρ_start_, and the interval of motion of the input link, i.e., angle P = |ρ_end_ − ρ_start_|, while the optimization variables of the lower eyelid are: the length of the input link R_0_R, the length of the output link OT, the angle of the input link in the initial position σ_start_, and the interval of motion of the input link, i.e., Σ = |σ_end_ − σ_start_|.

The eyes of the robot must fit in the space available in the head of the robot, so the bounds of the mechanism dimensions are given—Table 3 and Table 4 for the upper and lower eyelid, respectively.

The following variables are prescribed according to design requirements—the eyeball center is adopted as the coordinate system origin O(0,0,0). The axes of rotation of the input and output links are parallel to the y-axis, meaning **n_ρ_** = **n_σ_** = (0,1,0) and **n_θL_** = **n_θU_** = (0,1,0), respectively. The positions of the fixed points U_0_ and R_0_ (actuator positions) are U_0_ (−100,30,−15) and R_0_ (−100,30,−35), respectively; in the closed position θ_Uclosed_, point V coincides with the vertical yOz plane, while link OV is at a 60° angle relative to the y-axis, meaning **v_s_** = (−23.880,17.998,20.038). Furthermore, in the open position θ_Lopen_, point T coincides with the vertical yOz plane, while link OT is at a −60° angle relative to the y-axis, meaning $t_{s}=\left(0,\bar{\mathrm{OT}}\cos60{}^{\circ},-\bar{\mathrm{OT}}\sin60{}^{\circ}\right).$

According to the statements above, the dimensional optimal synthesis of the RSSR mechanism was conducted for the upper and lower eyelid, yielding the values shown in Table 5 and Table 6, respectively.

**Figure 12:**
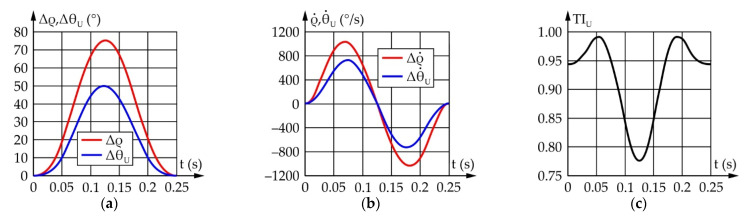
Upper eyelid mechanism: (**a**) Angular displacement of input and output link; (**b**) Angular speed of input and output link; (**c**) TI for single blink.

Figure 12 shows the results of the upper eyelid mechanism simulation conducted according to the values from Table 5. It should be noted that Δρ i Δθ_U_ represent the motion of the input links relative to their initial positions defined as ρ_start_ and θ_Uopen_, respectively.

According to Figure 12, for upper eyelid range of motion to be 50°, the actuator needs to rotate by 75.3°. The maximum angular speed of the upper eyelid equals 727.9°/s, with the required angular speed of the actuator being 1034.6°/s. The TI value changes from 0.62 to 0.98, which is satisfactory.

**Figure 13:**
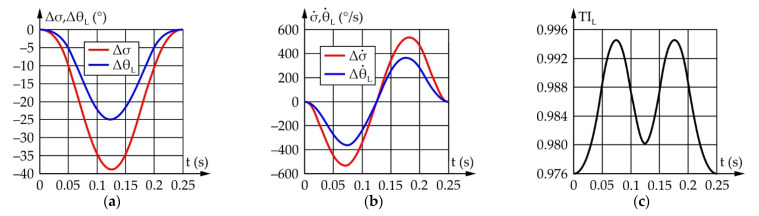
Lower eyelid mechanism: (**a**) Angular displacement of input and output link; (**b**) Angular speed of input and output link; (**c**) TI for single blink.

Figure 13 shows the results obtained by conducting a simulation of the lower eyelid mechanism according to the data from Table 6. It should be noted that Δσ and Δθ_L_ represent the movement of the input and output links relative to their initial positions defined as σ_start_ and θ_Lopen_, respectively.

According to Figure 13, for the lower eyelid to achieve a range of motion of 25°, the actuator must rotate by 38.9°. The maximum angular speed of the lower eyelid equals 353.4°/s, while the required angular speed of the actuator equals 535.9°/s. The TI value changes from 0.62 to 0.98, which is satisfactory.

#### 5.2.3. Mechanisms of Eyebrows

**Figure 14:**
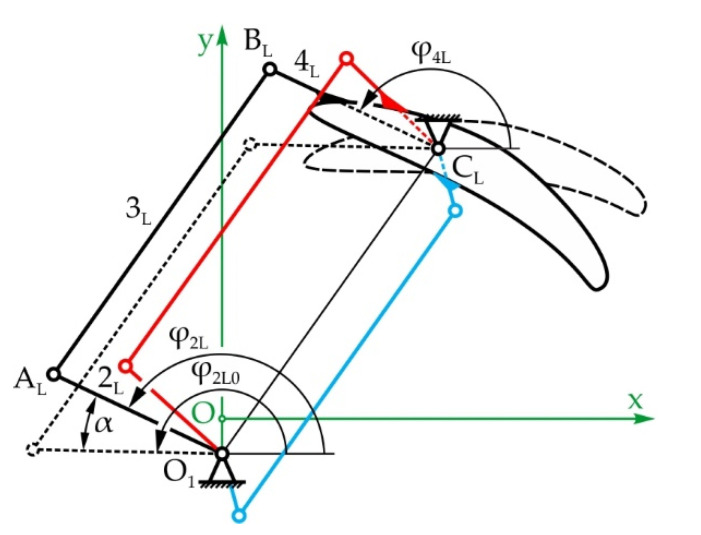
Left eyebrow rotation mechanism—potential solutions.

Figure 14 shows the simplest solution of the left mechanism, a parallelogram four-bar linkage with opposite links of equal lengths. This means that the position angles of the input and output links are equal, so φ_2L_ = φ_4L_. Additionally, the length of link 3_L_ is defined as well, and is equal to the distance between fixed points O_1_ and C_L_. The lengths of levers 2_L_ and 4_L_ must be equal to each other—however, their length is not unambiguously defined, as there is an infinite number of possible solutions.

Since link 2 consists of two levers 2_L_ and 2_R_ which are fixed to one another and therefore rotate together, if the left link rotates for angle α, the right one will as well (please see Figure 7). Considering that the left mechanism has a parallelogram configuration, the left eyebrow will rotate for that same angle α. Keeping in mind that the eyebrows should move symmetrically in regard to a vertical axis, it is evident that the right eyebrow must rotate for the angle −α. According to this, the design of the right four-bar linkage is considered to be the synthesis of a function generator, the solving of which requires the use of optimization methods:

(45) $$\varphi_{4R}-\varphi_{4R0}=-\alpha =-\left(\varphi_{2R}-\varphi_{2R0}\right)$$

The objective function is defined as the square of the difference between the rotation angles of the input and output links in regard to the initial—horizontal position:

(46) $$f\left(x\right)={\sum_{i}\left(\left(\varphi_{4\mathrm{Ri}}-\varphi_{4R0}\right)-\left(-\alpha_{i}\right)\right)}^{2}$$

where α_i_ = −20°, −19°,…, 0°,…, +19°, +20°, meaning the eyebrows rotate in regard to the horizontal position for ±20°. The adopted dimensions of the mechanism are as follows: the eyeball diameter is 60 mm, the PD is 96 mm, and the points around which the eyebrows rotate are C_R_ (−30,44) mm and C_L_ (30,44) mm, with the actuator being placed in point O_1_ (0,10). It should be noted that the dimensions of the eyeball and the PD were adopted from the MARKO robot, whose eyes and eyebrows are the subject being reconstructed in this paper.

The optimization variables are the lengths of the links r_2R_, r_3R_, and r_4R_, and the initial—neutral position angle of the input link φ_2R0_. In addition, the *i*-th position of the input link is expressed as:

(47) $$\varphi_{2\mathrm{Ri}}=\varphi_{2R0}+\alpha_{i}$$

Additionally, the mechanism must stay assembled and be efficient in all positions. The dynamic efficiency of the mechanism is defined by the transmission angle:

(48) $$\gamma_{R}=\varphi_{3R}-\varphi_{4R}$$

As the transmission angle grows, a larger part of the supplied power is spent on overcoming the work load, and less is spent on internal loads, making the mechanism more efficient. Small transmission angle values can cause the mechanism to jam. Due to this, the minimum value of the transmission angle is prescribed as γ_Rmin_ = 45°. Keeping in mind the available space in between the eyes (see Figure 6a), the minimum and maximum values of the input link angle φ_2R0_ are prescribed. Table 7 presents the minimum and maximum values of the optimization variables.

According to the statements above, the dimensional optimization synthesis of the right eyebrow mechanism was conducted and the obtained values are shown in Table 8.

Since the left eyebrow mechanism has a parallelogram configuration, and keeping in mind the dimensions of the right eyebrow mechanism, the lengths of links 2_L_ and 4_L_ are adopted as r_2L_ = r_4L_ = 10 mm, with the floating link length being calculated as the following:

(49) $$r_{3L}=\sqrt{{\left(x_{O1}-x_{\mathrm{CL}}\right)}^{2}-{\left(y_{O1}-y_{\mathrm{CL}}\right)}^{2}}=45.35\mathrm{mm}\;$$

**Figure 15:**
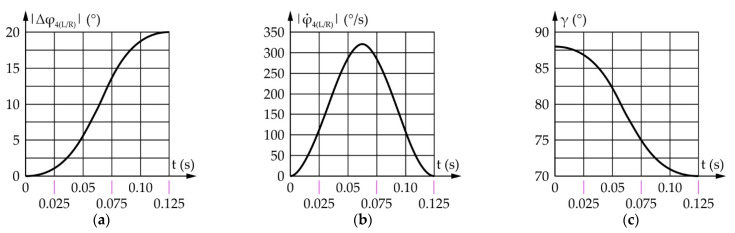
Eyebrow rotation mechanism: (**a**) Angular displacement of input and output link—absolute value; (**b**) Angular speed of input and output link—absolute value; (**c**) Transmission angle for the downward motion of the right eyebrow.

Figure 15 shows the results of the eyebrow rotaion mechanism simulation. It should be noted that |Δφ_4(L/R)_| represents the absolute value of the movement of output links 4_L_ and 4_R_ relative to their intial—horizontal position.

According to Figure 15, the range of motion, and the angular speeds of the eyebrows and the actuator are the same and equal, 20° and 320.0°/s, respectively. Since the transmission angle value depends on the side of the mechanism (left/right) and the direction of the eyebrow rotation (±), Figure 15c shows one of four cases of the transmission angle change. The values of the transmission angle in all four cases stay within the prescribed bounds, i.e., from 67° to 110°.

**Figure 16:**
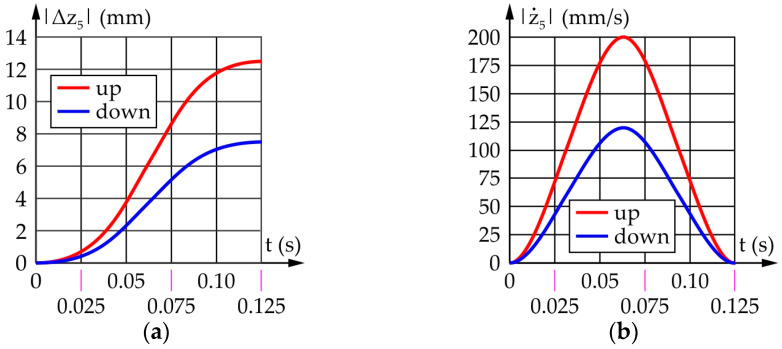
Eyebrow raising/lowering mechanism: (**a**) Angular displacement of output link—absolute value; (**b**) Angular speed of output link—absolute value.

Figure 16 shows the results obtained from a motion simulation of the eyebrow raising/lowering mechanism output link. It should be noted that |Δz_5_| represents the absolute values of the displacement of the output link relative to its initial position.

According to Figure 16a, the total vertical stroke of the eyebrow equals 20 mm, of which 12.5 mm is the raising and 7.5 mm the lowering. Figure 16b shows the maximum speeds of the output link of the mechanism during reflexive movement of the eyebrow during a fear response—the raising speed is 200.0 mm/s, while the lowering speed is lower and equals 120 mm/s, which is comparable to [177].

### 5.3. Summary

Table 9 summarizes and presents the results of the structural and dimensional synthesis of the eyeball, eyelid, and eyebrow driving systems. It should be noted that the angular speed of the input link of the eyebrow raising/lowering mechanism directly depends on the parameters of the spindle drive mechanism, such as the diameter of the threaded shaft, the type and pitch of the thread, the angle of the thread, and number of starts of the thread—see Figure 6b, making it easy to calculate.

According to Table 9, the relationship between the change in position of the input/output links of the eyeball and eyelid mechanisms was defined to ascertain the effect on the control system. Due to the structure of the eyeball mechanism rotating the eyeball in the horizontal plane, as well as the mechanisms for the rotation and translation of the eyebrows, the relationship between the relative movements of the output/input links is linear in all three cases, meaning that Δψ = Δβ, Δφ_4(L/R)_ = Δφ_2(L/R)_, and Δz_5_ = *c* actuator displacement, where *c* = const.

**Figure 17:**
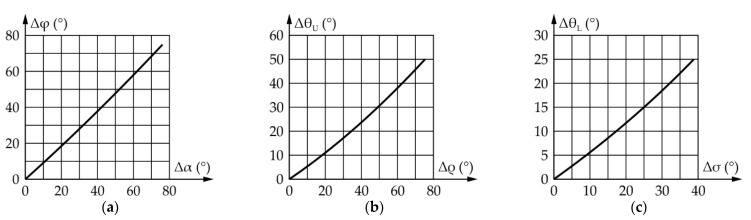
The relationship between the change in position of the input/output mechanism links: (**a**) Δφ/Δα for the eyeball rotation in the horizontal plane; (**b**) Δθ_U_/Δρ for the upper eyelid rotation; (**c**) Δθ_L_/Δσ for the lower eyelid rotation.

Figure 17a shows the relative change in position of the eyeball during the rotation in the vertical plane Δφ with regard to the relative change in position of the mechanism input link Δα. The relationship is very nearly linear, the nonlinearity—the largest deviation from a straight line connecting the first and last point on the graph, equals only 2.38%. Figure 17b shows the relative change in position of the upper eyelid Δθ_U_ with regard to the relative change in position of the mechanism input link Δρ, while Figure 17c shows the relative change in position of the lower eyelid Δθ_L_ with regard to the relative change in position of the mechanism input link Δσ. The nonlinearity was determined to be 5.86% for the upper eyelid and 4.94% for the lower eyelid, making the relationship in both cases close to linear.

According to the statements above, it is concluded that the determined relationships are very close to linear, which is very favorable for control system purposes. In the following chapters, the structure of the control system is explored, and possible components are suggested for use in the eyeball, eyelid, and eyebrow mechanism control system. Additionally discussed is the structure of a servo controller meant to control a single actuator within the suggested control system.

## 6. Control System Architecture

**Figure 18:**
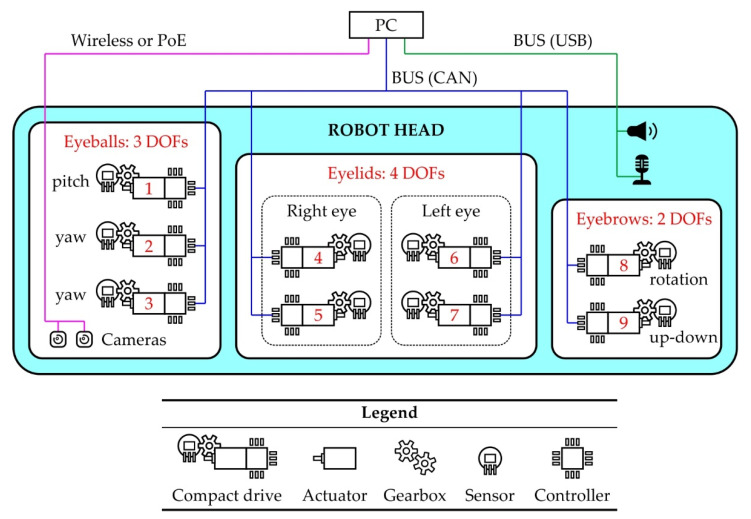
Hierarchical overview of the control system.

Figure 18 shows the hierarchy of the robot eye control system. The movement of the eyeballs, eyelids, and eyebrows is enabled by the joint action of 9 actuators, of which 3 are for the eyeballs, 4 for the eyelids, and 2 for the eyebrows. Relatively simple and efficient actuator implementations are miniature DC motors. In order to achieve the desired kinematic parameters of the eye output links, all DC motors require precise and sophisticated control. An embedded personal computer (PC), a single-board computer or a high-performance microcontroller, is at the top of the hierarchical structure and synchronizes the entire system by sending commands to all subordinate control units. This component also directly controls the audio output (sound signals, speech). Digitized audio input for speech recognition can be assigned to this system. Additionally, the images captured by the cameras, placed inside the eyeballs, are processed by the computer at the top of the hierarchical structure.

According to Figure 18, compact drive systems for actuating the mechanisms of the eyes, eyebrows, and eyelids have been proposed. The eyeball is actuated via three actuators. Actuator 1 is common to both eyeballs allowing simultaneous pitch movements (vertical saccades), while actuators 2 and 3 allow independent yaw movements of eyeballs in the same or opposite directions (horizontal saccades and focusing objects—stereovision). The movement of the upper and lower eyelids is completely independent and is enabled by the four actuators, of which actuators 4 and 6 are for the upper eyelids, while actuators 5 and 7 are for the lower eyelids. The remaining actuators enable independent rotation and translation of the eyebrows. Therefore, actuator 8 allows both eyebrows to rotate simultaneously, but in opposite directions, while actuator 9 allows both eyebrows to be raised simultaneously. By combining different movements and positions of the eyeballs, eyelids, and eyebrows, it is possible to generate a wide range of non-verbal facial expressions of the robot.

A reasonably simple and efficient solution is the use of DC motors with a built-in planetary gearhead (with one or more stages) and an integrated incremental encoder for actuation of the eye’s moving parts. For position detection, in addition to the incremental encoder, an absolute position sensor can be used. The elimination of the zero position sensor, which enables the adjustment of the initial position of the system, is the advantage of using the absolute position sensor.

**Figure 19:**
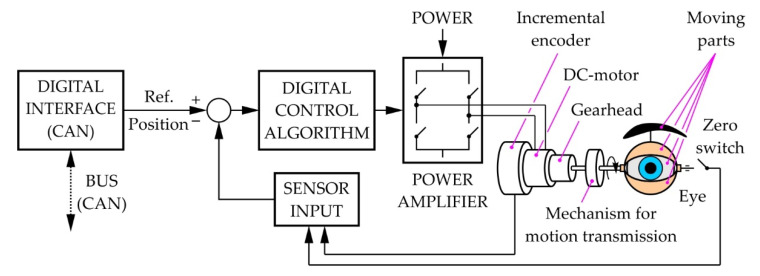
Internal structure of a single servo controller.

Figure 19 shows the structure of a slave servo controller. It controls a single actuator which directly affects 1 DOF within the system, assuming the actuator is a DC motor type. Via a digital interface—for example, controller area network (CAN), the master controller sets the required target positions or position change profiles that the controlled element should achieve during a given time. The assigned value is set as the reference input of the control algorithm. It should be noted that the control algorithm is implemented on a microcontroller or digital signal processor of appropriate performance, performing its function based on monitoring the current position of the DC motor shaft via an incremental encoder. Power is transmitted to the motor by an amplifier—implemented as a bridge driver, which is directly controlled by the control algorithm. When initializing the servo controller, the zero position sensor (switch or optical sensor) allows the system to be brought to a known initial position.

It should be noted that the motor and planetary gearhead must be selected in such a way that, at the available voltage, the motor can achieve an angular speed slightly higher than the one sufficient to achieve the fastest required movement of the mechanical part it drives. In addition, communication between individual controllers within the system can be achieved using a robust communication network, such as a CAN bus.

### Summary

In order to realize the desired motion of the mechanical system of the eyeballs, eyelids and eyebrows, the structure of the control system is given. For the actuation of the mechanisms, compact drive systems which include an actuator (motor), planetary gearhead, sensor, and motor controller, are proposed. The optimal variant, from a control perspective, would be a DC motor with a suitable planetary gearhead and absolute position sensor or incremental encoder. In order to control each individual actuator, one servo controller is provided according to the proposed structure. The improvement of the proposed control system structure is possible, e.g., using wearable blindness-assistive devices (sensors, global positioning system (GPS), light detection and ranging (LIDAR), and RGB-D camera), and simultaneous localization and mapping (SLAM) technology [178].

## 7. Non-Verbal Communication Effectiveness

Humans as social beings strive for interaction with other subjects, and may interpret absence of emotional expression as indifference—it is thus desirable that robotic characters express emotional states when communicating with humans [179]. It was established that humans are able to perceive and understand emotional states expressed by a robot even from a relatively small number of moving points on its face [180], which suggests further that feelings are something that a human eye looks for on another subject’s (even a robot’s) face.

To determine the level at which the suggested eye and eyebrow design enables the robot to convey non-verbal emotions, an experiment was designed and conducted. Here, the purpose was to measure to which extent this set of eyes and eyebrows was capable of successfully expressing emotions to a set of human subjects. Six basic emotions (surprise, fear, disgust, anger, happiness, and sadness) were chosen as relevant for the experiment—these basic emotions were shown to be universally identifiable through Ekman’s work on measuring facial movement during expressions of emotions [181]. In order to break down every facial manifestation of an emotion, Ekman designed a comprehensive facial action coding system (FACS). This system was designed for interpreting common emotional expressions by identifying specific muscular activity that produces momentary changes in facial appearance—these specific movements are called action units (AUs) and they may be coded as “upper eyelid raiser” or “inner eyebrow movement”, for example [182].

Previous research into emotion expression by robot faces utilize these aforementioned AUs, while still acknowledging that facial features of any robot are extremely sparse with highly constrained motion compared to a human face [183]. In a robot’s upper part of the face, there are typically only a few DOFs, but still, previous research has shown that there is a set of minimal features for human-like facial expressions that are effective in communicating emotions [180]. Based on Ekman’s seminal work [184], as well as from research related to robot faces [185,186], this study started by defining AU sets for the six basic emotions, focusing only on the eye and eyebrow movements (see Table 10).

### 7.1. Description of the Video Clips

**Figure 20:**
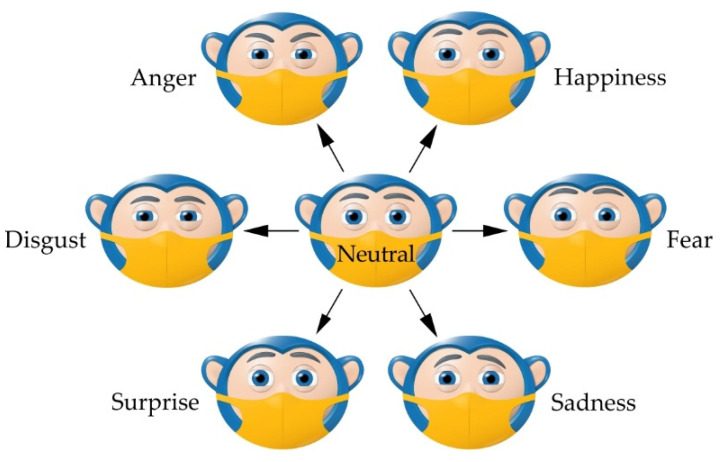
Human-like robot MARKO and his facial expressions.

Based on the existing robot MARKO [187], a 2D image of his face was designed, but also altered from the original model by covering the robot’s mouth and nose area with a face mask. This alteration was made for two reasons: first, to focus participants’ attention to the upper half of the face which was in line with the research goal. Second, to mask the part of the face which was not movable, thus not being effective in expressing an emotion—since the mouth and the nose are generally a significant part of non-verbal expression [181,184], presenting them as static while other parts of the face are moving would lead to incongruent expressions and potentially confusing stimuli. Additionally, the global COVID-19 pandemic experienced in 2020/2021 and the resulting usage of face masks in everyday communication inspired the research team to present the robot with a mask covering its nose and mouth. According to the data from Table 10, Figure 20 presents the six basic facial expressions of the human-like robot MARKO: anger, disgust, surprise, happiness, fear, and sadness.

**Figure 21:**
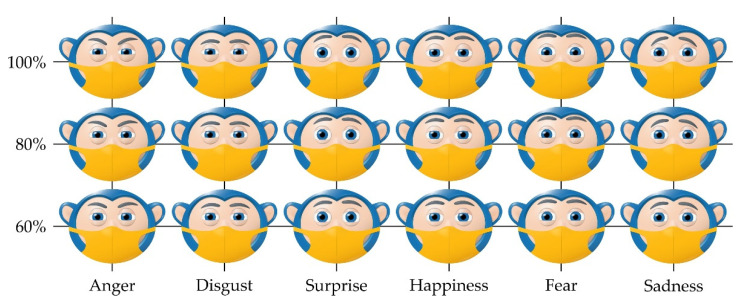
Six different facial expressions of the robot showing an emotion with different levels of intensity.

In order to increase reliability by taking multiple measurements of the same stimulus, it was decided that every emotion should be expressed by the robot’s face to a participant three times, mixed randomly with expressions of other emotions [186]. However, it was decided not to present the identical stimuli three times for every emotion; rather, different intensities of the emotion in question were presented, by expressing 60%, 80%, and 100% of every AU, thus allowing us to also check if the intensity of an expression had any role in emotion recognition effectiveness [179]. Figure 21 shows the 6 different facial expressions of the robot with varying intensity.

From the neutral face and the still images of robot MARKO expressing the six emotions, video clips were created. Similarly to previous relevant studies [180,186], each video clip consisted of 5 points: (i) starting point, where the robot’s face was in the neutral position—a total of 3 s, (ii) transition period, where the robot shows progress towards an emotion articulation—a total of half a second, (iii) facial expression of an emotion—a total of 3 s, (iv) transition period, where the robot reverts back to the neutral position—a total of half a second, and (v) ending point, where the robot’s face is shown still again in the neutral position—a total of 3 s. Therefore, the total duration of each video clip is 10 s.

### 7.2. Experiment Procedure

The experiment was conducted in a laboratory setting, with controlled light and sound, and without any significant distractions. The experiment was conducted in an improvised laboratory space at the university office. Each participant was seated in front of a 23′′ computer screen, at a 2 m distance.

The goal of the experiment was to determine to which extent the structural design of the eyes and eyebrows is capable of emotional expression, in a way that conveys the intended emotion to human observers. Since the eye is an important non-verbal actor in emotional exchange in interpersonal communication, it is of relevance to measure to which extent our model is effective in expressing basic emotions.

For this experiment, 51 participants were recruited, all of them being university students at the bachelor level. The participants ranged from 18 to 27 years old (mean age 21.57), 29 were females, and 22 males. The participants were not aware of the goal of the study, and reported no prior experience with similar models or research studies. 

After giving informed consent and a short introduction about what to expect during the experiment, each participant was shown 18 video clips with the model expressing an emotion. For each subject, the video clips were presented. Each participant was presented with all 18 video clips, presented in random order, with the constraint that the same intended emotion was never presented twice in a row. The participants were allowed to take as long as they wished to complete every task, but were not allowed to have the same video played again.

After each video clip, the subjects were presented with a short printed facial expression identification (FEI) instrument [180], consisting of three questions. Question #1 was a simple task to identify the shown emotion, by choosing one term from an alphabetized list of six basic emotion labels that they believed best suited what they have seen. Next, the participants were presented with Question #2—they were asked to rate the degree to which the emotion was present—the strength of expression—on a scale of 0 (not at all) to 6 (extremely high), similar to [138,180,183,188]. Question #3 was then asked, allowing the participants (but not requiring) them to select one or more “other expressions” they thought the model might be displaying beyond the primary one, identified in the first question, if desired—similar to [180]. In subsequent sections, we refer to “main accuracy” based on the single answer from Question #1, and “other accuracy” when including answers from both Questions #1 and #3.

### 7.3. Results

The completed printed FEI questionnaires were fed into a data matrix, and analyzed with IBM’s SPSS software, version 23. Globally, the study participants’ first guess was effective in 45.8% of cases—main accuracy was achieved in 420 cases out of 918. The study participants made a second guess in 25.7% of the cases, and that second guess was effective in 20.8% of occurrences—in 49 cases out of 236 where there was a correct second guess. Combined, the participants were successful in recognizing expressed emotions in 51.1% of cases, either on the first try or on the second try.

Table 11 presents a confusion matrix where emotions identified by the study participants (in rows, counting only their first guess) and emotions expressed by the robot (columns) are cross-tabulated. The cells in this table contain percentages of the matches or mismatches between the two variables, where the diagonal direction from top left to bottom right presents the matches (grayed cells), and all the other table cells present mismatches. From this table, it is evident that the expressions of anger and sadness were successfully identified to a large extent (92.8% and 83.7%, respectively). The expression of surprise was correctly identified in half of the cases (51.6%), while it was frequently confused with fear (27.5%). The expression of disgust was correctly identified in one third of the cases (35.5%), being frequently confused with anger (28.9%). Expression of happiness was seldom correctly identified (6.7%), frequently being mistaken for disgust (34.2%), and surprise (31.5%). The expression of fear was also rarely correctly identified (4.6%), mostly being mistaken either with surprise (46.4%), or with happiness (38.6%).

A separate analysis of the first identified emotion for every video clip, for each of the three levels of expression intensity, does not indicate that the level of expression intensity plays any significant role in the identification of the emotion—participants were opting for the same emotions regardless of the intensity of the movement. Even more interestingly, emotions of surprise and fear were better identified when shown with 60% and 80% of intensity, than when expressed with 100% of intensity (see Table 12).

**Figure 22:**
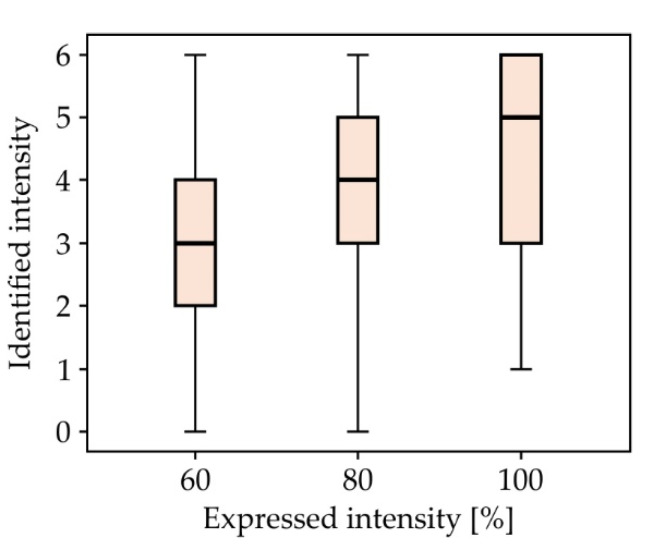
Box and Whisker plot of identified intensity of expressed emotions (0–6) for all three levels of expression by the robot face (60%, 80%, and 100).

Although the level of expression intensity does not play a role in the kind of perceived emotion, that level is still noticed by the participants. A weak but highly significant positive correlation between the expressed intensity of an emotion by the robot and the perceived emotion intensity by the participants was observed, using the Spearman’s rho correlation coefficient due to the ordinal measurement levels of the FEI scales (Spearman’s rho = 0.304, *p* = 0.000). This finding shows that the participants had some success in identifying the intensity of an expressed emotion: for an emotion that was expressed at 60%, on a 0–6 scale, participants identified intensity with a median of 3; for an emotion that was expressed at 80%, participants identified intensity with a median of 4, and for an emotion that was expressed at 100%, participants identified intensity with a median of 5, as shown on a Box and Whisker plot in Figure 22. This finding is even more interesting if observed separately for each expressed emotion: for the three emotions that were most successfully recognized in the first attempt (anger, sadness, and surprise), the correlation coefficients were even higher and were interpreted as “moderate” (Spearman’s rho = 0.570, *p* = 0.000, Spearman’s rho = 0.452, *p* = 0.000, and Spearman’s rho = 0.462, *p* = 0.000, respectively).

Participants’ gender did not play a role in the effectiveness of emotion identification: although there was a slight difference observed between females’ and males’ percentage of main accuracy (46.9% and 44.2%, respectively), this difference was not significant (Phi = 0.027, *p* = 0.409), nor was there any significant difference observed in the other accuracy between the two genders (Phi = 0.023, *p* = 0.479). Running these analyses for every emotion separately yielded similar results.

Aiming to determine if there was any effect of training on the accuracy of emotion identification, we have split the dataset into three thirds, based on the order of video clips that were shown to the participants, and observed the percentage of main accuracies in each third of the experiment. However, there were no significant differences to report.

### 7.4. Summary

The presented results show that, globally, the proposed structural design of the robot eyes is capable of effectively expressing emotions of anger and sadness to a high extent, which is in line with previous studies, and partially for the emotion of surprise; expressions of disgust, happiness, and fear are poorly identified, being frequently misinterpreted as other emotions. Emotions of anger and sadness are most specific in this setup since the eyebrows take extreme positions regarding their vertical position and the position of their outer ends; this “uniqueness” of expression of these two emotions is in line with the “overlap” rule from a previous study that notices that “the fewer DOFs in a given emotion that overlap with other emotions, the better the recognition will be for that emotion” [186] (p. 4580). This rule is also evident in the case of surprise—although the vertical eyebrow movement reaches its full extent, the outer end does not move significantly and uniquely for this emotion, which explains the result that it was properly identified only in half of the cases. Limitations of effective expression of other emotions are, similarly, coherent with the “coverage” rule defined by the same authors, which states that “the greater the proportion of required action units in a given emotion that can be mapped to DOF in the robot, the better the recognition will be for that emotion” [186] (p. 4580). It is documented by Ekman [181] that some emotions need movements on other parts of the face for proper expression, which were not considered in this study: surprise requires the “jaw drop” movement; fear is accompanied by the “fear mouth” movement (and some-times lacks any eyebrow movement at all); disgust is primarily expressed with the unique mouth movement and the nose wrinkles; happiness is mostly shown through the lip movement and the nasolabial fold that runs down from the nose to the outer edge beyond the lip corners. Additionally, expressions of fear, surprise, and happiness are not so far apart from each other when the eye and eyebrow are observed—movements in other parts of the face are crucial for valid interpretation of these emotions [184]. Especially, the emotion of fear is one of the most complex expressions to produce in terms of the number and control of muscles used, besides considering the fact that its infrequency of use in daily life might also be a factor in the difficulty people have in identifying it [189].

## 8. Results and Discussion

This section summarizes the results and contains: (i) the comparison of the proposed mechanical system with the kinematics of the human eye, (ii) the advantages of the adopted mechanisms and their reconfigurability, and (iii) the ability of the proposed mechanism to generate facial expressions.

### 8.1. Capability of the Mechanical System 

The mechanical system consists of three subsystems which enable the independent motion of the eyeballs, eyelids, and eyebrows. Due to its structure, the eyeball mechanical system is able to generate all of the motions of a human eye, which is the main condition for the realization of binocular function of the artificial robot eyes, as well as for stereovision. Saccades are significant for rapid movements, while vergence movements allow the eyes to focus on objects. Aside from reflexive movements, it is also important to realize smooth pursuit movements whose generation and quality directly depend on the structure of the adopted mechanisms and their joints—the friction and backlash in the joints should be as low as possible. Contrarily, initiating movement would cause a jerk which can negatively affect the stability of the visual image, especially since the face, object, and surrounding recognition cameras would be located in the eyes of the robot. From a kinematic standpoint, the mechanical systems of the eyeballs, eyelids, and eyelashes are very capable at mimicking the human eye. Table 13 shows the comparison between the kinematic parameters of the human eye and the parameters of the proposed mechanical system.

It should be noted that the eyebrow movements are complex and depend on which part of the eyebrow is being actuated, as well as in which direction. It should also be noted that human eyebrows cannot be rotated, only raised and lowered. During reflexive eyebrow movement due to a fear response, the eyebrows move together with the upper eyelids with a much higher speed of 25 mm/s, which was found in the available literature. The amplitude of the eyebrow raising heavily depends on and decreases with age. After searching the available literature, the range of motion of the lower eyelid could not be found, so it was estimated according to the fact that the range of motion of the upper eyelids is approximately two times larger than that of the lower ones—of course, the range of motion of the upper eyelids directly depends on the type of blink (see Section 2). All of the kinematic parameters found in Table 13 refer to the extreme values.

### 8.2. Adopted Mechanisms

Most of the adopted mechanisms driving the mechanical systems of the eyeballs, eyelids, and eyebrows are linkage mechanisms, with a spindle drive mechanism being adopted for raising the eyebrows. Linkage mechanisms allow for a wide range of working speeds, are highly reliable, have low backlash, and are simple to manufacture and assemble; while the spindle drive mechanism enables the transformation of rotational into translational motion, has a wide range of possible pitches and speeds, also has low backlash, high reliability, and is simple to implement. Low backlash enables high positioning accuracy which further enables high precision and repeatability of movements, which is key. Linkage mechanisms can have different structures and link shapes, making them easy to optimize. The spherical motion of the eyeball is easiest to implement with spatial linkage mechanisms. Keeping in mind the limited space in the robot’s head, due to the many electronic components placed there, the most favorable solution for the transmission of power and motion are spatial linkage mechanisms. The motion of the mechanism output link is defined by the axis around which it rotates—for example, the eyelids rotate around the y-axis. By using spatial linkage mechanisms, the designer has the option to choose the axis of rotation of the input link, for example, around the x-, y- or z-axis, which allows the design of the mechanism to be adapted to the available space in the head. Another convenience is that the mechanism links can be made using 3D printing technology, which results in parts with very low mass. This would significantly lower the inertial loads present in the mechanism due to high acceleration values, especially during reflexive movements.

**Figure 23:**
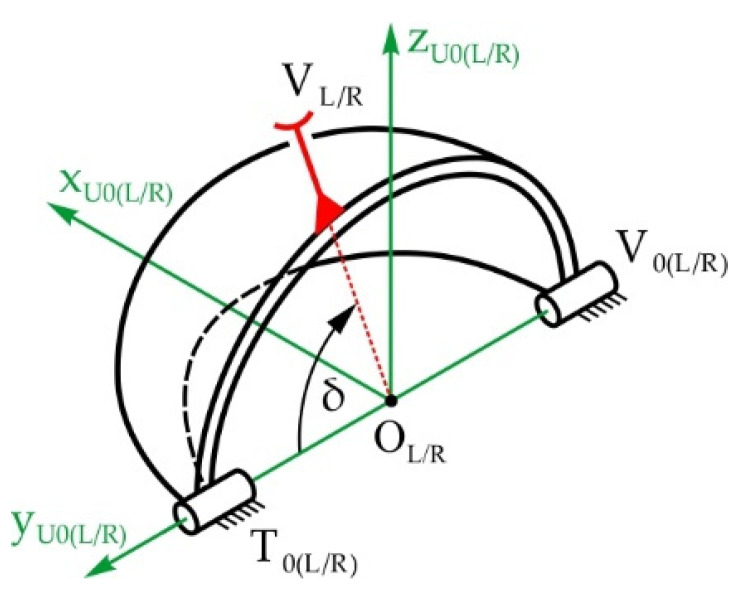
Upper eyelid—the position of link OV with regard to angle δ.

Figure 23 shows the output link of the upper eyelid mechanism. Link OV must rotate around the y-axis, but it can be placed in different positions. Due to this, it is interesting to determine the possible range of its placement without changing the kinematics of the upper eyelid. The constructive parameter—angle δ, can vary from 40–80°. It cannot be less than 40° due to collisions with the side of the face, and it cannot be over 80° due to collisions with the eyeball. From a design point of view, this information is very significant which is why the reconfigurability of the mechanism was examined.

**Figure 24:**
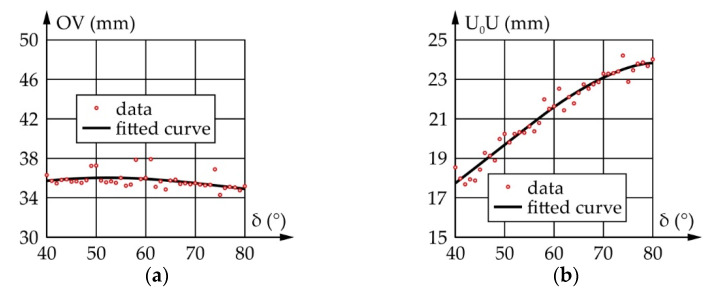
The variation of the upper eyelid geometric parameters with regard to angle δ: (**a**) Output link length; (**b**) Input link length.

**Figure 25:**
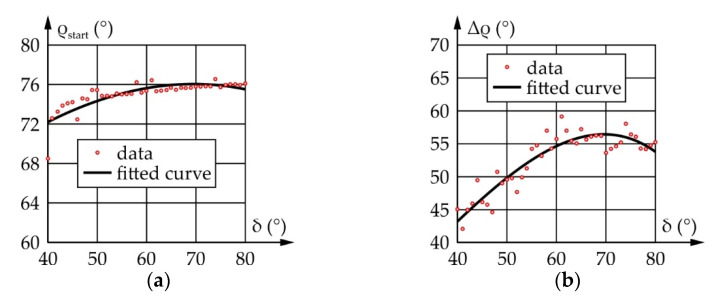
The variation of the upper eyelid geometric parameters with regard to angle δ: (**a**) Initial position of input link; (**b**) Interval of motion for input link.

Based on the process shown in Section 5.2, dimensional synthesis of the upper eyelid was conducted for each possible value of angle δ, and the results can be seen in Figure 24 and Figure 25. It is possible to assemble the mechanism for every value of angle δ within the interval 40–80°. The dimensions of the other links remain within the prescribed bounds, meaning the kinematic behavior remains unchanged. These data can be acquired for the lower eyelid in a similar way.

### 8.3. Non-Verbal Communication

This study aimed to determine if the proposed structural design of the robot eyes and eyebrows was capable of effectively expressing emotions to human subjects. This aim was pursued by exposing study participants to a series of short video clips where the robot MARKO was expressing basic emotions identified by Ekman. Recognizability of Ekman’s basic expressions is a common test used to gauge the abilities of an expressive robot face [190]. The recorded accuracies are seen as a good sign especially since only video clips of the robot were shown—physically present robots are perceived more persuasively, and result in better user performance than their visually presented counterparts [191]; physical presence often seems crucial for good perception of emotional information conveyed by a robotic agent [192]. It was interesting to observe that the emotion of disgust was inconsistently identified in this study, since this emotion is frequently omitted from these kinds of experiments, due to its specific expression that also includes a nose movement [189,192]. The level of intensity of emotional expression was properly identified to a significant degree, especially for emotions of anger, sadness, and surprise, showing that, at least for these emotions, the level of movement can express the intensity of an emotion. The fact that the level of intensity of emotional expression did not play a significant role in the accuracy of emotion identification is in line with previous research that showed that even when an emotion is presented with 50% intensity in a robot’s face, human subjects were still able to identify robotic facial expressions [180]. The fact that females and males were equally successful in emotion identification is not in line with previous research, which showed that females were more accurate when identifying emotions [193,194]. It should be noted that in the last two years, interest has risen for the recognition of emotions on faces equipped with face masks [195,196,197,198,199,200,201]. Additionally, it should be stated that the study participants were all similar in age—being university students, and without any reported relevant health issues, which may pose a limitation in the generalizability of the obtained results, since it has been shown that children and the elderly may have different abilities in recognizing facial expressions when compared to young adults [202,203,204], and that people with certain mental health issues experience facial emotion recognition deficits when compared to control groups [205,206,207]. This is especially important if the proposed solution is to be implemented in the context of healthcare, where specific cohorts are usually treated.

## 9. Conclusions

This paper shows the structure of a mechanical system for robot eyes with a total of 9 DOFs, as well as its ability to allow the robot to generate non-verbal emotional content, which is a key characteristic of socially interactive robots. The mechanical system enables independent movement of the eyeballs, eyelids, and eyebrows, and consists of three subsystems: (i) the mechanical system of the eyeballs, (ii) the mechanical system of the eyelids, and (iii) the mechanical system of the eyebrows. The mechanical system of the eyeballs has 3 DOFs allowing for simultaneous pitch and independent yaw movements of the eyeballs. Due to its structure which, among other things, allows for the placement of a camera within the eyeball, the mechanical system is able to reproduce all of the movements of a human eye, which is of great significance for the realization of binocular function of artificial sight, as well as for stereovision. The mechanical system of the eyelids has 4 DOFs, enabling independent rotation of each eyelid, while the mechanical system of the eyebrows has 2 DOFs, enabling the simultaneous raising of both eyebrows, as well as the rotation of both eyebrows in opposite directions. From a kinematic standpoint, the mechanical systems of the eyeballs, eyelids, and eyebrows are able to generate movements sufficiently similar to natural human ones—the types of movements, the ranges of motion, and the angular speeds, which is of great significance for the generation of facial expressions and non-verbal communication of robots in a natural, intuitive, and transparent way.

It should be noted that the relationship between the motion of the input/output links were examined for each mechanism, to ascertain its influence on the control system—the obtained relationships were all very close to linear, which is very favorable from the standpoint of the control system. Due to the joint structure, all of the mechanisms ensure both low friction and low backlash, which is important for initiating movement without jerks, as well as for highly accurate positioning which ensures high precision and repeatability. The structure of a control system for the eyeballs, eyelids, and eyebrows was proposed with the goal of realizing the motion of the mechanism’s output links so that it is in accordance with the kinematic principals of the human eye. Compact drive systems which encompass the actuator—motor, reducer, sensor, and motor controller, were proposed to drive the mechanisms. The most favorable solution for controlling the system is a combination of a DC motor with an appropriate reducer and an absolute position sensor or an incremental encoder. The structure of a servo controller for each specific motor was proposed as well.

Finally, the success of the mechanical system depended on how capable it was to enable the robot to generate facial expressions, which is why an experiment was conducted. For this purpose, the 2D face of existing robot MARKO was used, covered with a face mask to aid in focusing the participants on the eye region. The participants rated the efficiency of the robot’s non-verbal communication after watching short video clips. The proposed structural design of the robot eyes was capable of effectively expressing emotions of anger and sadness to a high extent, and only partially the emotion of surprise. Expressions of disgust, happiness, and fear were poorly identified and were frequently misinterpreted as other emotions. To make happiness and fear more recognizable, the face would need to be fully uncovered, thus necessitating the existence of lips and their precise positioning, while the emotion of disgust requires specific motion of the nose and forehead.

Further research will encompass the physical realization of each of the described mechanical systems, their implementation and experimental examinations meant to determine the kinematics and the efficiency of the non-verbal communication. Furthermore, also planned is the development and realization of eyes with a positive CT, which is a feature of female eyes. Further research should also encompass emotion expressions with other parts of the robot face, in order to determine which combination of facial movements produces the best results.

## 10. Patents

The driving mechanisms of the mechanical systems of the eyeballs, eyelids, and eyebrows described in this paper are patent pending.

## Figures and Tables

**Table 1:** Lower and upper bounds of optimization variables of the eyeball.

	K_0_K (mm)	OL (mm)	α_start_ (°)	A (°)
lower	15	15	50	50
upper	25	25	90	90

**Table 2:** Dimensions of the RSU leg rotating the eyeball around the y-axis.

K_0_K (mm)	KL ^1^ (mm)	OL (mm)	α_start_ (°]	A (°)
24.00	78.78	23.18	68.36	76.20

^1^ The length of the floating link KL is unambiguously defined by the prescribed and optimized geometric parameters.

**Table 3:** Upper eyelid—lower and upper bounds of optimization variables.

	U_0_U (mm)	OV (mm)	ρ_start_ (°)	P (°)
lower	15	34	220	50
upper	25	40	240	90

**Table 4:** Lower eyelid—lower and upper bounds of optimization variables.

	R_0_R (mm)	OT (mm)	ρ_start_ (°)	Σ (°)
lower	15	34	125	25
upper	25	40	145	45

**Table 5:** The dimensions of the RSSR mechanism driving the upper eyelid.

U_0_U (mm)	UV ^1^ (mm)	OV (mm)	ρ_start_ (°)	P (°)
21.64	93.03	35.99	228.94	75.33

^1^ The length of the floating link UV is unambiguously determined by the prescribed and optimized values of the geometric parameters.

**Table 6:** The dimensions of the RSSR mechanism driving the lower eyelid.

R_0_R (mm)	RT ^1^ (mm)	OT (mm)	σ_start_ (°)	Σ (°)
23.93	118.04	35.60	129.50	38.94

^1^ The length of the floating link RT is unambiguously determined by the prescribed and optimized values of the geometric parameters.

**Table 7:** Right eyebrow mechanism—lower and upper bounds of optimization variables.

	r_2R_ (mm)	r_3R_ (mm)	r_4R_ (mm)	φ_2R0_ (°)
lower	10	40	10	50
upper	20	50	20	90

**Table 8:** The dimensions of the right eyebrow rotation mechanism.

r_2R_ (mm)	r_3R_ (mm)	r_4R_ (mm)	φ_2R0_ (°)
10.99	40.00	10.99	70.79

**Table 9:** Structural and kinematic parameters of the eyeball, eyelid, and eyebrow driving mechanisms.

Parameter	Eyeballs		Upper Eyelids	Lower Eyelids	Eyebrows	
Movement type	Vertical saccades (around y-axis)	Horizontal saccades (around z-axis)	Blink (around y-axis)	Blink (around y-axis)	Rotation (around x-axis)	Raising/Lowering (along z-axis)
Mechanism type	2 RSU legs interconnected with RRRR parallelogram mechanism, total 1 DOF	2 × 2 planar 4-bar linkages, independent for each eyeball, total 2 DOFs	2 independent RSSR linkages, total 2 DOFs	2 independent RSSR linkages, total 2 DOFs	7-link planar mechanism for independent and/or simultaneous rotation and translation movements, total 2 DOFs	
Output link displacement	75° (from −30° to 45°)	90° (from −45° to 45°)	±50° (opening/closing)	±25° (opening/closing)	±20° (left/right)	+12.5/−7.5 mm (up/down)
Movement duration	0.2 s (up/down)	0.2 s (right/left)	0.125/0.125 s (opening/closing)	0.125/0.125 s (opening/closing)	0.125/0.125 s (left/right)	0.125/0.125 s (up/down)
Max. output speed	769.1°/s	899.5°/s	727.9°/s	353.4°/s	320.0°/s	200.0/120.0 mm/s (up/down)
Input link displacement	76.2°	90°	75.3°	38.9°	20°	spindle drive mechanism ^1^
Max. input speed	770.4°/s	899.5°/s	1034.6°/s	535.9°/s	320.0°/s	

^1^ The input parameters directly depend on the parameters of the spindle drive mechanism (see Figure 6b).

**Table 10:** Sets of AUs for every emotion intended for expression.

Emotion	Upper Eyelid	Lower Eyelid	Eyebrow Vertically	Eyebrow Outer End	Gaze
Anger	lowered partially	raised until covering part of the iris	lowered to a full extent	pointed upwards to a full extent	direct
Disgust	lowered partially	raised until covering part of the iris	lowered to a full extent	straight	direct
Surprise	raised until sclera is visible above	in neutral position	raised to a full extent	pointed downwards slightly	direct
Happiness	lowered slightly	raised slightly	raised slightly	straight	direct
Fear	raised until sclera is visible above the iris	raised until covering part of the iris	raised to a full extent	straight	direct
Sadness	raised slightly	raised slightly	raised to a full extent	pointed downwards to a full extent	lowered

**Table 11:** Confusion matrix of the recognition rates of identified emotions, shown as percentages of total occurrence of an expressed emotion.

		Emotion Expressed by the Robot					
		Anger	Disgust	Surprise	Happiness	Fear	Sadness
Emotion Identified ^1^	Anger	92.8	28.9	0.0	3.4	2.0	0.0
	Disgust	5.2	35.5	2.6	34.2	6.5	2.6
	Surprise	1.3	9.9	51.6	31.5	46.4	1.3
	Happiness	0.0	7.2	5.9	6.7	38.6	1.3
	Fear	0.7	11.8	27.5	12.8	4.6	11.1
	Sadness	0.0	6.6	12.4	11.4	2.0	83.7

^1^ Emotion identified by the study participants in their first guess.

**Table 12:** Numbers of correct emotion identification occurrences with each of the three levels of intensity of the robot’s facial expression. Note: the total number of participants was 51.

Expressed Intensity of Emotion (%)	Expressed Emotion					
	Anger	Disgust	Surprise	Happiness	Fear	Sadness
60	47 (92.1%)	14 (27.4%)	30 (58.8%)	4 (7.8%)	2 (3.9%)	40 (78.4%)
80	45 (88.2%)	22 (43.1%)	31 (60.8%)	4 (7.8%)	5 (9.8%)	44 (86.3%)
100	50 (98.0%)	18 (35.3%)	18 (35.3%)	2 (3.9%)	0 (0%)	44 (86.3%)

**Table 13:** Comparison of the kinematic parameters of the human eye and the proposed mechanical system—extreme values.

Comparison	Eyeball				Eyelids				Eyebrows			
	Pitch		Yaw		Upper		Lower		Rotation		Raising/Lowering	
	ROM (°)	AS (°/s)	ROM (°)	AS (°/s)	ROM (°)	AS (°/s)	ROM (°)	AS (°/s)	ROM (°)	AS (°/s)	ROM (mm)	S (mm/s)
Human	75	800	90	800	±45	1100	±20	N/A	no	no	+15/(N/A)	25/(N/A)
PSD	75	769.1	90	899.5	±50	727.9	±25	353.4	±20	320.0	+12.5/−7.5	200.0/120.0

Note: PSD—proposed structural design; ROM—range of motion; AS—angular speed; S—speed; N/A—not available.
